# Perfecting and extending the near-infrared imaging window

**DOI:** 10.1038/s41377-021-00628-0

**Published:** 2021-09-24

**Authors:** Zhe Feng, Tao Tang, Tianxiang Wu, Xiaoming Yu, Yuhuang Zhang, Meng Wang, Junyan Zheng, Yanyun Ying, Siyi Chen, Jing Zhou, Xiaoxiao Fan, Dan Zhang, Shengliang Li, Mingxi Zhang, Jun Qian

**Affiliations:** 1 State Key Laboratory of Modern Optical Instrumentations, Centre for Optical and Electromagnetic Research, College of Optical Science and Engineering, International Research Center for Advanced Photonics, Zhejiang University, 310058 Hangzhou, China; 2 Intelligent Optics & Photonics Research Center, Jiaxing Institute of Zhejiang University, 314000 Jiaxing, Zhejiang Province China; 3 State Key Laboratory of Advanced Technology for Materials Synthesis and Processing, Wuhan University of Technology, 430070 Wuhan, China; 4 Key Laboratory of Reproductive Genetics (Ministry of Education), Department of Reproductive Endocrinology, Women’s Hospital, Zhejiang University School of Medicine, 310006 Hangzhou, China; 5 College of Pharmaceutical Sciences, Soochow University, 215123 Suzhou, China

**Keywords:** Biophotonics, Imaging and sensing

## Abstract

In vivo fluorescence imaging in the second near-infrared window (NIR-II) has been considered as a promising technique for visualizing mammals. However, the definition of the NIR-II region and the mechanism accounting for the excellent performance still need to be perfected. Herein, we simulate the photon propagation in the NIR region (to 2340 nm), confirm the positive contribution of moderate light absorption by water in intravital imaging and perfect the NIR-II window as 900–1880 nm, where 1400–1500 and 1700–1880 nm are defined as NIR-IIx and NIR-IIc regions, respectively. Moreover, 2080–2340 nm is newly proposed as the third near-infrared (NIR-III) window, which is believed to provide the best imaging quality. The wide-field fluorescence microscopy in the brain is performed around the NIR-IIx region, with excellent optical sectioning strength and the largest imaging depth of intravital NIR-II fluorescence microscopy to date. We also propose 1400 nm long-pass detection in off-peak NIR-II imaging whose performance exceeds that of NIR-IIb imaging, using bright fluorophores with short emission wavelength.

## Abstract (web summary)

Moderate light absorption by bio-tissue is conducive to the imaging performance. The second near-infrared window is perfected as 900–1880 nm, and 2080–2340 nm is proposed as the third near-infrared window.

## Graphical abstract

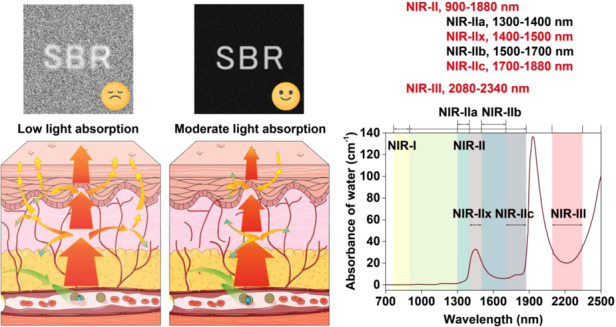

## Introduction

Fluorescence imaging has been widely utilized in medical practices. With the deepening of understanding of the interaction between light and bio-tissue as well as the cost decline of detection technique, the fluorescence imaging wavelength as a whole is red-shifted constantly from visible range to near-infrared (NIR) region^1,2^. The energy loss when light propagates in the biological media could be blamed on the absorption attenuation and the scattering disturbance. The absorption loss determines whether we could catch the signals while the scattering signals always reduce the definition of images. In addition, excessive light absorption in bio-tissue might induce tissue injury. The autofluorescence from some biomolecules is always mingled with the useful signals and eventually becomes the background of taken images. Thus, the deep-rooted beliefs that light absorption and scattering are totally harmful to fluorescence catching urge most researchers to chase a perfect window with minimal photon absorption and scattering for bioimaging. Due to the generally accepted less photon scattering, the fluorescence bioimaging in the second near-infrared window (NIR-II) gives admirable image quality, especially when deciphering the deep-buried signals in vivo^3–7^. Nowadays, NIR-II fluorescence imaging has already guided complicated liver-tumor surgery in clinic^8^. However, the constructive role of light absorption, to some extent, seems to be ignored. The final presentation of high-quality images even makes the overstated positive effect of scattering suppression by lengthening wavelength more convincing since the absorption simultaneously is considered to attenuate the signals. As a matter of fact, some works have revealed absorption-induced resolution enhancement in the scattering media due to the depressing of long optical-path background signals^9,10^. Yet how to take full advantage of light absorption to select a suitable fluorescence imaging window remains unspecified.

The definition of the NIR-II window has been always limited to 1000–1700 nm, prompting the launch of various NIR emitters with the peak emission wavelength beyond 1000 nm^11,12^, and even beyond 1500 nm (NIR-IIb region, 1500–1700 nm)^13–15^. Some existing and developing fluorophores with peak emission below 1000/1500 nm but bright emission tail beyond 1000/1500 nm, meanwhile, are also well-suited for NIR-II/NIR-IIb fluorescence imaging^16–21^, including some excellent probes in aggregates^21–24^. It must be admitted that the design and synthesis of bright and long-wavelength NIR emitters are still full of challenges nevertheless. Besides, the positive role of light absorption mentioned above in NIR-II fluorescence imaging could undermine the privilege of ultralong emitters; in other words, it is not an inevitability that the longer the imaging wavelength, the better the imaging performance. Therefore, preparation of wavelength-tunable NIR-II fluorophores with stable brightness is still not easily available, but of great significance for us to search for the optimum imaging window, even exceeding the NIR-IIb region (e.g., beyond 1700 nm). In addition, organic agents are always considered well biocompatible but it is indeed hard to equip the organic dyes with both long wavelength and strong emission. Since the emission tailing usually occupies a few of the whole, the agents were directly applied to detection in some given long-pass (LP) spectral regions and perform high-contrast imaging^25,26^. However, the experience guides us to avoid the light absorption peak of biomolecules (such as water) when determining the imaging window. The best LP band for imaging, therefore, still needs to be carefully verified, considering the positive influence of absorption.

Microscopic examination of the tiny bio-structure is always constructive to the understanding of biological processes and diagnosis, as well as treatment for certain diseases. Fluorescence wide-field microscopy in the NIR-II region has shown its excellent strength of deep deciphering in bio-tissues of rodents^7,27–29^ and even nonhuman primates^19,30^. The user-friendly imaging mode with high temporal resolution could assist operators in monitoring the dynamic process in real time such as blood flow. However, defocusing signals and scattering light are often collected along with the targeted information, and thus become the strong background, consequently reducing the image contrast. Some advanced NIR-II microscopic imaging techniques, such as confocal^15,27,28,31^, and light-sheet microscopy^32^, aim to counteract the image background via adjusting the collection, and excitation pattern. However, the pinhole-introduced scanning confocal microscopy wastes the useful signals inevitably and lengthens the imaging duration compared with area-detection microscopy. The light-sheet excitation always places great demands on the transparency of the sample. In this way, microscopy with the high spatio-temporal resolution, deep penetration, and easy operation is still urgently needed.

Herein, we used the Monte Carlo method to simulate the NIR photon propagation in bio-tissue and innovatively proposed the well-performance imaging in 1400–1500, 1700–1880, and 2080–2340 nm, which were defined as near-infrared IIx (NIR-IIx), near-infrared IIc (NIR-IIc), and the third near-infrared (NIR-III) window, respectively. Lead sulfide (PbS) quantum dots (QDs) have exhibited the advantages of high fluorescence brightness and tunable emission wavelength^33–35^ for noninvasive high-performance in vivo imaging in the NIR-II window^11,15,36^. We designed and synthesized a series of PbS/CdS core-shell quantum dots (CSQDs), and then hydrated them with polyethylene glycol (PEG). Assisted by the bright QDs with peak emission wavelength at ~1100, ~1300, and ~1450 nm, we found the detection regions around the absorption peaks of water always provide vastly improved image quality, and thus the definition of the NIR-II window was further perfected as 900–1880 nm. The 1400–1500 nm which was defined as the NIR-IIx region was proved to provide more superior fluorescence images than the NIR-IIb region. The in vivo NIR-IIx fluorescence microscopic cerebral vasculature imaging with the convenient optical setup but the strongly suppressed background was conducted with admirable image contrast and the imaging depth reached ~1.3 mm, which represented the deepest in vivo NIR-II fluorescence imaging in mice brain to date. Considering the emission of substantial fluorophores peaking shorter than 1400 nm but holding bright emission tailing, we proposed an effective usage of 1400 nm LP collection with better performance than the NIR-IIb region. We believed these results are pretty crucial for the further development of NIR fluorescence imaging.

## Results

### Simulation and discussion on the near-infrared fluorescence imaging

**Fig. 1:**
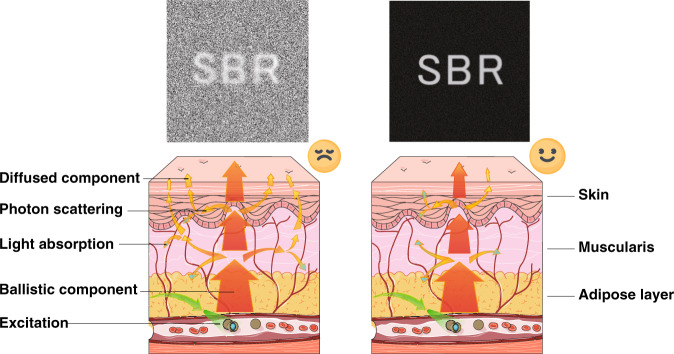
Schematic diagram of light propagation in tissue. The propagation of excited ballistic and diffused emission photons in the bio-tissue with small (left) and moderate (right) light absorption and the resulting signal-to-background ratios (SBRs) of fluorescence imaging.

**Fig. 2:**
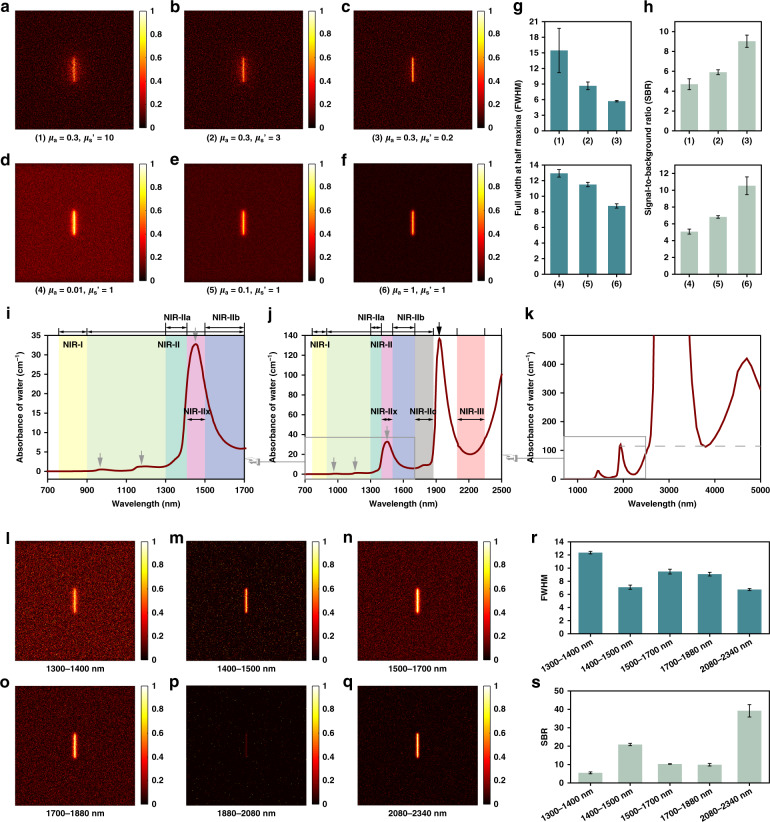
The simulation results of NIR bio-tissue imaging via the Monte Carlo method. Images of a line source through a bio-tissue of 1-mm thickness with scattering anisotropy factor (*g*) of 0.9, absorption coefficient (*μ*_a_) of 0.3 mm^−1^, and varying reduced scattering coefficient (*μ*_s_*’*) (**a**) *μ*_s_*’* = 10 mm^−1^, (**b**) *μ*_s_*’* = 3 mm^−1^ and (**c**) *μ*_s_*’* = 0.2 mm^−1^. Images of a line source through a bio-tissue of 1 mm thickness with g of 0.9, *μ*_s_*’* of 1 mm^−1^ and varying absorption coefficient (**d**) *μ*_a_ = 0.01 mm^−1^, (**e**) *μ*_a_ = 0.1 mm^−1^, and (**f**) *μ*_a_ = 1 mm^−1^. The (**g**) FWHM and (**h**) SBR analyses of the samples in (**a**–**f**). The light absorption spectra of water within (**i**) 700–1700 nm^38^, (**j**) 700–2500 nm^38^, and (**k**) 900–5000 nm^39^. The gray arrows pointed out the absorption peaks and the gray dashed line in (**k**) represented the absorbance at 1930 nm. Equivalent images of a line source through a bio-tissue of 1 mm thickness in (**l**) 1300–1400 nm, (**m**) 1400–1500 nm, (**n**) 1500–1700 nm, (**o**) 1700–1880 nm, (**p**) 1880–2080 nm, and (**q**) 2080–2340 nm. The (**r**) FWHM and (**s**) SBR analyses of the samples in (**l**–**q**).

General cognition of the NIR-II window guides us to emphasize the scattering depression with the increase of wavelength but underestimate the constructive effect of absorption. As a matter of fact, the light absorbers would preferentially deplete the multiply scattered photons in propagation in theory since scattered photons have longer path lengths through the biological medium than ballistic photons (see Fig. 1). Monte Carlo method was utilized to simulate the propagation of photons in biological tissues here^37^ and the imaged line sample is shown in Supplementary Fig. S1. The scattering depression would benefit the transmission of ballistic photons and the effective collection. With the decrease of the reduced scattering coefficient (*μ*_s_*’* = 10 mm^−1^ in Fig. 2a; *μ*_s_*’* = 3 mm^−1^ in Fig. 2b; *μ*_s_*’* = 0.2 mm^−1^ in Fig. 2c) but the same absorption coefficient (*μ*_a_) of 0.3 mm^−1^, the image became clearer (see Fig. 2a–c). Then the *μ*_s_*’* was set to 1 mm^−1^, and *μ*_a_ was 0.01, 0.1, and 1 mm^−1^ in Fig. 2d, e, and f, respectively. It could be seen that the full width at half height (FWHM) declined (see Fig. 2g) and the signal-to-background ratio (SBR) grew significantly (see Fig. 2h) with the decrease of the scattering and the light absorption in tissue could inhibit the strong background and enhance the spatial resolution (see Fig. 2g) as well as imaging contrast (see Fig. 2h).

The light absorption spectra within 700–5000 nm of water^38,39^, which is the most important component of organisms, are shown in Fig. 2i–k. As is widely known to all, the 360–760 nm is defined as the visual region, the NIR region thus starts with 760 nm. The traditional bioimaging window is usually located in the first near-infrared (NIR-I) region, which is ranging from 760 to 900 nm^40,41^. The light absorption by water substantially improves beyond 900 nm, compared with the NIR-I window. The gray arrows pointed out the absorption peaks at ~980, ~1200, ~1450, and ~1930 nm, where the spectral regions around the peaks would improve the imaging quality in principle. Because of the absorption peak at ~980 nm, 900–1000 nm should not be excluded from the NIR-II window for bioimaging. The imaging in 1400–1500 nm has not long been recognized, but the high light absorption within this band, which is called as NIR-IIx region here, is no longer the barrier in the NIR-II region, as long as the fluorescent probes possess enough brightness to resist the attenuation by water. At present, the photoresponse of the classic InGaAs detector limited the optical imaging beyond 1700 nm, thus the NIR-II window was defined as no more than 1700 nm. Because of the similar absorption and scattering properties, we believed that 1700–1880 nm possessed comparable imaging quality with the NIR-IIb imaging and defined 1700–1880 nm as the NIR-IIc region. It could be calculated that the peak absorption intensity at ~1930 nm is near e^100^ times higher than the peak at ~1450 nm for every 1 cm of transmission, thus the useful signals with the wavelengths near 1930 nm would be almost impossible to detect in deep tissue. Hence, the NIR-II window was perfected as 900–1880 nm. Over the absorption “mountain” peaking at ~1930 nm, the region of 2080–2340 nm, which was newly considered as the third near-infrared (NIR-III) region here, becomes the last high-potential bio-window in general since the water absorption of light beyond 2340 nm keeps stubbornly high (see Fig. 2k). The borderline wavelengths of the NIR-III window (2080 nm and 2340 nm) were determined due to the similar light absorbance to that at 1450 nm. In addition, it should be noted that the emission detection near the light absorption peak provides excellent imaging quality in the area-detection fluorescence imaging, however, the window with light absorption is unsuited for excitation due to the photo-thermal damage in general.

In order to verify the above hypotheses, we then simulated the photon propagation in 1300–1400 nm (NIR-IIa), 1400–1500 nm (NIR-IIx), 1500–1700 nm (NIR-IIb), 1700–1880 nm (NIR-IIc), 1880–2080 nm, and 2080–2340 nm (NIR-III) window (see Fig. 2l–q), taking the absorption spectrum of water and the scattering property of skin into consideration. The average of multiple simulation results (see Supplementary Fig. S2) within one NIR region was considered as the equivalent result of the window. As shown in Fig. 2r, s, except for the extremely intense depletion in 1880–2080 nm (see Fig. 2p), rising light absorption and falling photon scattering both made positive contributions to the precise imaging. Interestingly, the NIR-IIx imaging showed the best FWHM and SBR in the whole NIR-II region. Compared with the NIR-IIb region, the production of scattering photons was less but the depletion of scattering photons (determined by absorption) dropped at the same time in the NIR-IIc region. Altogether, the NIR-IIc imaging also performed well with good definition and high SBR. Furthermore, since the subcutaneous adipose tissue absorbs more light in the NIR-IIc region, the NIR-IIc imaging holds greater potential in obese organisms^42^. The simulation results are displayed in Supplementary Fig. S3. Although the light absorption by water decreases, the absorption of subcutaneous adipose tissue rises when the imaging window is red-shifted from the NIR-IIb to the NIR-IIc region (Supplementary Fig. S3a). The advantages of the NIR-IIc imaging through subcutaneous adipose tissue could be recognized from the simulation results in Supplementary Fig. S3b, c. Compared with the imaging in the NIR-IIb window, sharper FWHM and higher SBR could be obtained in the NIR-IIc imaging through the adipose tissue (Supplementary Fig. S3d). Significantly, the newly defined NIR-III imaging possessed the greatest potential for bioimaging owing to the large but proper absorbance and the exceedingly low photon scattering, according to the results from Monte Carlo simulation. We believed the NIR-IIc and NIR-III imaging would be achieved sooner or later with the rapid development and further popularization of NIR detectors as well as the emergence of bright agents.

### 900–1000 nm should be included in the NIR-II window

The NIR-II region has long been defined as 1000–1700 nm in the academic world^43^. Meanwhile, many InGaAs-based products enable imaging from 900 nm to 1700 nm and this range has also been a typical band of shortwave infrared in the industrial field. The boundary of these two similar regions has been ambiguous. In particular, 900–1000 nm seems to have not been recognized as a part of the NIR-II region by most research groups. According to the advantages of moderate light absorption mentioned above, 900–1000 nm might be a good imaging window considering that there is an absorption peak at ~980 nm.

**Fig. 3:**
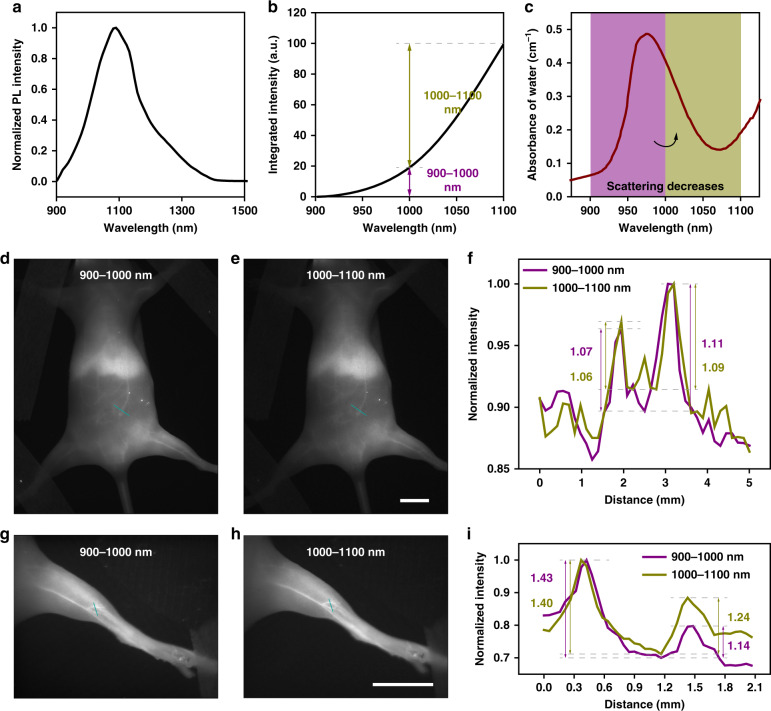
Comparison of in vivo fluorescence imaging of mice in 900–1000 and 1000–1100 nm. **a** The emission spectrum of PEGylated 1100-PbS/CdS QDs in water. **b** The integrated emission intensity of PEGylated 1100-PbS/CdS QDs in water. The value of the ordinate represents the integrated emission intensity between 900 nm and the ordinate corresponding wavelength. The value difference on the ordinate represents the total emission intensity in the corresponding spectral (abscissa) range. **c** The absorbance of water within 800–1200 nm^38^. The whole-body imaging of the same mouse in (**d**) 900–1000 nm and (**e**) 1000–1100 nm. **f** Cross-sectional fluorescence intensity profiles along the indigo lines of the blood vessel in (**d**, **e**). The numbers show the SBRs. The hind limb imaging of the same mouse in (**g**) 900–1000 nm and (**h**) 1000–1100 nm. **i** Cross-sectional fluorescence intensity profiles along the indigo lines of the blood vessel in (**g**, **h**). The numbers show the SBRs. Scale bars: 10 mm.

To examine the imaging window in the NIR-II region, the PbS QDs were developed, whose brilliant fluorescence intensity and stability would facilitate the long-time monitoring and tracking in vivo^44,45^. Besides, the broad excitation with narrow and symmetry emission of PbS QDs could enable multiplex imaging in vivo^46,47^, compared with other NIR-II-emitting fluorophores. First, the PbS/CdS QDs emitting at ~1100 nm were synthesized, which were named as 1100-PbS/CdS QDs. The absorption and emission spectra of 1100-PbS/CdS QDs in tetrachloroethylene are displayed in Supplementary Fig. S4. As shown in Fig. 3a, b, the PEGylated 1100-PbS/CdS QDs showed emission in NIR-II region, and the integrated emission intensity of the 1100-PbS/CdS QDs in 900–1000 nm was weaker than that in 1000–1100 nm. It could be seen that though the 900–1000 nm window possessed higher photon scattering, the light absorption by water in this region was also stronger, compared with the band of 1000–1100 nm (see Fig. 3c). The transmission electron microscopic (TEM) image (see Supplementary Fig. S5a) and the dynamic light scattering (DLS) result (see Supplementary Fig. S5b) identified the uniformly spherical QDs with a mean hydrodynamic diameter of 29.9 nm. The zeta potential was measured as −2.0 mV (see Supplementary Fig. S5c).

In vivo fluorescence imaging of mice was then conducted under the excitation of a 793 nm CW laser. As shown in Fig. 3d, e, despite the dominant intensity proportion within 1000–1100 nm of the PEGylated 1100-PbS/CdS QDs, the whole-body imaging quality in 900–1000 nm was not significantly worse than that in 1000–1100 nm post intravenous injection (1 mg mL^−1^, 200 μL). In contrast, the quantitative results in Fig. 3f even gave us strong evidence about the better SBRs of 1.07 and 1.11 in 900–1000-nm imaging, while the SBRs of the same vessels were 1.06 and 1.09 in 1000–1100-nm imaging. By adjusting the field of view, the whole hind limb could be clearly presented (Fig. 3g, h), giving the further comparison of imaging quality between 900–1000 and 1000–1100 nm. The contrasts in the images were demonstrated to be level-pegging, which is shown in Fig. 3i. That is to say, though the photon scattering decreases with the increase of wavelength, the strong absorption at ~980 nm could actually suppress the image background. As shown in Supplementary Fig. S6, the same conclusion could be drawn by the comparison of the whole-body mice imaging in the two windows after intravenous injection with the reported fluorescent probe emitting at ~1000 nm^18^. Besides, the distinct advantages of imaging in 900–1000 nm were verified by other groups compared with the imaging in 700–900 nm^48^. In view of the same detection mechanism and decent imaging performance between the spectral region of 900–1000 and 1000–1100 nm, the former should not be excluded from the NIR-II imaging window.

### The rising light absorption by water from ~1300 nm “turns on” the promising stage for NIR-II fluorescence imaging

**Fig. 4:**
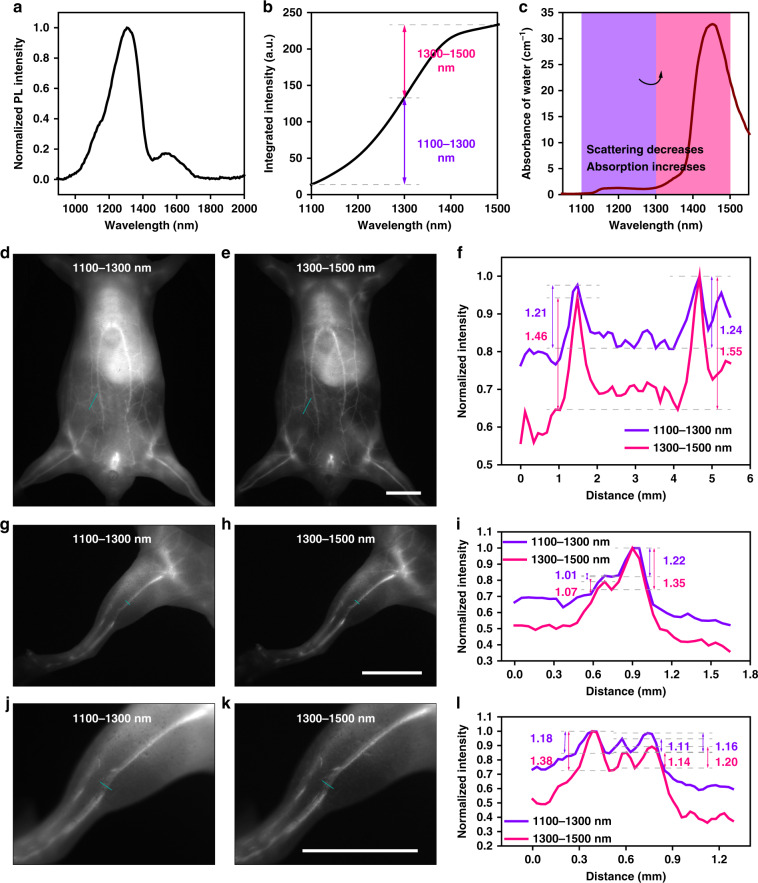
Comparison of in vivo fluorescence imaging of mice in 1100–1300 and 1300–1500 nm. **a** The emission spectrum of PEGylated 1300-PbS/CdS QDs in water. **b** The integrated emission intensity of PEGylated 1300-PbS/CdS QDs in water. The value difference on the ordinate represents the total emission intensity in the corresponding spectral (abscissa) range. **c** The absorbance of water within 1000–1600 nm^38^. The whole-body imaging of the same mouse in (**d**) 1100–1300 nm and (**e**) 1300–1500 nm. **f** Cross-sectional fluorescence intensity profiles along the indigo lines of the blood vessel in (**d**, **e**). The numbers show the SBRs. The hind limb imaging of the same mouse in (**g**) 1100–1300 nm and (**h**) 1300–1500 nm. **i** Cross-sectional fluorescence intensity profiles along the indigo lines of the blood vessel in (**g**, **h**). The numbers show the SBRs. The hind limb imaging of the same mouse at a larger magnification in (**j**) 1100–1300 nm and (**k**) 1300–1500 nm. **l** Cross-sectional fluorescence intensity profiles along the indigo lines of the blood vessel in (**j**, **k**). The numbers show the SBRs. Scale bars: 10 mm.

The PbS/CdS CSQDs emitting at ~1300 nm were next synthesized for the comparison of imaging in 1100–1300 nm and 1300–1500 nm, whose absorption and emission spectra are shown in Supplementary Fig. S7. After PEGylation, the 1300-PbS/CdS QDs were spherical with a hydrodynamic size of 35.2 nm from the TEM (see Supplementary Fig. S8a) and DLS (see Supplementary Fig. S8b) results, and the zeta potential was measured as −2.8 mV (see Supplementary Fig. S8c). The normalized and integrated emission spectra of the PEGylated 1300-PbS/CdS QDs are presented in Fig. 4a, b and the calculated fluorescence intensity in 1300–1500 nm was marginally lower than that in 1100–1300 nm, which might be blamed on the light absorption by water. It was apparent from Fig. 4c that the shifting from 1100–1300 to 1300–1500 nm not only decreased the photon scattering but also significantly heightened the light absorption, which would lead to a remarkable improvement in the background suppression.

After intravenous injection (1 mg mL^−1^, 200 μL), the whole-body vessel imaging of mice could be conducted under the excitation of a 793-nm CW laser. As displayed in Fig. 4d, e, the image background decreased significantly when shifting the spectral window from 1100–1300 to 1300–1500 nm. After calculation, the SBRs showed uplifts over 20 percent (see Fig. 4f). The hind limb vessel imaging was then performed in the two bands and the high-contrast advantage in 1300–1500-nm imaging was demonstrated once again (see Fig. 4g–i). When further enlarging the magnification, the adjacent three fine vascular structures could be still distinguishable with low background in 1300–1500 nm (see Fig. 4j–l) and each capillary exhibited the better SBRs of 1.38, 1.14, and 1.20 than those in 1100–1300 nm (1.18, 1.11, and 1.16). Typically, fluorescence bioimaging beyond 1300 nm, which included both NIR-IIa (1300–1400 nm) and NIR-IIb (1500–1700 nm) regions, has been already verified much more outstanding than that conducted between 900 and 1300 nm (in the short wavelength region of NIR-II region). It could be seen that the 200-nm redshift induced scattering depression and the surge in the absorption in especial contributed to the background attenuation. Then we injected the 1100-PbS/CdS QDs into other mice and conducted the 900–1100-nm and 1100–1300-nm imaging. However, Supplementary Fig. S9 shows that just the 200-nm redshift from 900–1100 to 1100–1300 nm did not similarly improve the imaging quality. It seemed that the substantial growth of light absorption but not the decrescent photon scattering governed the start of the promising stage for NIR-II fluorescence imaging.

### The optimum NIR-II imaging window locates around the intense absorption peak at ~1450 nm

**Fig. 5:**
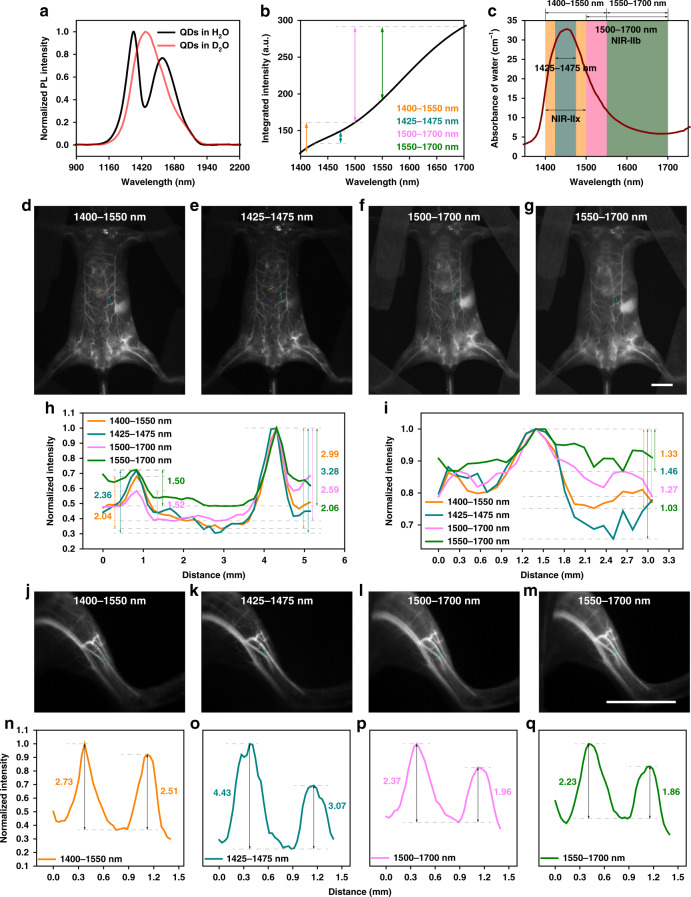
Comparison of in vivo fluorescence imaging of mice in 1400–1550, 1425–1475, 1500–1700, and 1550–1700 nm. **a** The emission spectra of PEGylated 1450-PbS/CdS QDs in water and heavy water. **b** The integrated emission intensity of PEGylated 1450-PbS/CdS QDs in water. The value difference on the ordinate represents the total emission intensity in the corresponding spectral (abscissa) range. **c** The absorbance of water within 1300–1800 nm^38^. The whole-body imaging of the same mouse in (**d**) 1400–1550 nm, (**e**) 1425–1475 nm, (**f**) 1500–1700 nm, and (**g**) 1550–1700 nm. Cross-sectional fluorescence intensity profiles along (**h**) the indigo lines and (**i**) brown lines of the blood vessel in (**d**–**g**). The numbers show the SBRs. The hind limb imaging of the same mouse in (**j**) 1400–1550 nm, (**k**) 1425–1475 nm, (**l**) 1500–1700 nm, and (**m**) 1550–1700 nm. **n**–**q** Cross-sectional fluorescence intensity profiles along the indigo lines of the blood vessel in (**j**–**m**). The numbers show the SBRs. Scale bars: 10 mm.

The intense absorption peak of water locating in the NIR-II region has been eschewed for its strong signal loss and potential thermal damage. To objectively evaluate the fluorescence imaging with collection around 1450 nm, the 1450-PbS/CdS QDs were synthesized whose absorption and emission spectra could be found in Supplementary Fig. S10. Plainly, the TEM (see Supplementary Fig. S11a) and DLS (see Supplementary Fig. S11b) results of PEGylated 1450-PbS/CdS QDs demonstrated a uniform spherical shape with an average hydrodynamic size of 35.2 nm and the zeta potential of −3.8 mV (see Supplementary Fig. S11c). The hydrogen bond and the O–H covalent bond in water make the absorption spectrum present multiple characteristic peaks in the infrared region. When the protium in water is replaced by deuterium, the molecular weight increases, and the characteristic absorption peak wavelength at ~1450 nm is red-shifted to ~1970 nm. The normalized photoluminescence spectra of PEGylated 1450-PbS/CdS QDs in hydrogen oxide (water) and deuterium oxide (heavy water) are exhibited in Fig. 5a. It was apparent that the intense absorption at ~1450 nm by water induced a large depression in the fluorescence spectrum, while the spectrum of the QDs in heavy water, where the corresponding absorption peak was red-shifted, restored the original emission characteristics. After integration, it was noticeable that the emission in water within 1425–1475 nm was depleted seriously (see Fig. 5b). Figure 5c directly shows the light absorption within 1300–1800 nm. The 1300–1400 and 1500–1700 nm were called as NIR-IIa and NIR-IIb regions, respectively, where the fluorescence imaging was proved with excellent quality. We now define the neglected region of 1400–1500 nm as the NIR-IIx window, which has long been considered unsuitable for imaging owing to the highest absorbance at ~1450 nm in the NIR-II region.

Under the excitation of a 793 nm CW laser, the whole-body vessel imaging of mice was conducted in 1400–1550, 1425–1475, 1500–1700, and 1550–1700 nm. It is worth noting that the 1425–1475 nm possesses a bandwidth of 50 nm around the absorption peak at ~1450 nm, which highlights the contribution of absorption by water; the band of 1400–1550 nm broadens the detection around 1450 nm with a bandwidth of 150 nm; The 1500–1700 nm is the classic NIR-IIb region with existing best performance; The 1550–1700 nm is the chosen region with the lowest photon scattering compared with the above three bands. As shown in Fig. 5d–g, the vessel imaging in 1425–1475 nm possessed the lowest imaging background. It is interesting to note that increasing the imaging wavelength did not make the performance as better as expected, since the absorption peak was located at ~1450 nm. Comparing the imaging in the four regions, the photon scattering in 1550–1700 nm is theoretically the lowest but the region with high expectations gave the worst image contrast. Meanwhile, the imaging in 1425–1475 nm provided the best SBRs of 2.36 and 3.28, and with the imaging region shifted farther away from the absorption peak, the SBRs became worse (2.04 and 2.99 in 1400–1550 nm, 1.52 and 2.59 in 1500–1700 nm, 1.50 and 2.06 in 1550–1700 nm), which is shown in Fig. 5h. On account of the accumulation of QDs in the liver and spleen, the strong background would blur the vessels above the two bright organs. However, as shown in Fig. 5i, the SBRs were calculated as 1.33, 1.46, 1.27, and 1.03 respectively in 1400–1550-, 1425–1475-, 1500–1700-, and 1550–1700-nm images, which revealed that the absorption-induced background attenuation could improve the definition effectively. The hind limb imaging with larger magnification was also conducted in the four regions. It could be seen in Fig. 5j–m that, the closer the imaging window is to the peak absorption, the lower the imaging background. As shown in Fig. 5n–q, the SBRs of the two tiny vessels were measured as 2.73 and 2.51 in 1400–1550 nm, 4.43 and 3.07 in 1425–1475 nm, 2.37 and 1.96 in 1500–1700 nm, 2.23 and 1.86 in 1550–1700 nm, which further confirmed the positive contribution of the absorption. In addition, the superiority of the window near the absorption peak at ~1450 nm still existed even if the typical NIR-IIb emissive 1600-PbS/CdS QDs^15^ were utilized for the in vivo imaging (see Supplementary Fig. S12). NIR-IIb fluorescence imaging has long been regarded as the most promising NIR-II fluorescence imaging technique due to the suppressed photon scattering until then, but our results now proved greater contribution of rising absorption than the decrescent scattering and the NIR-IIx fluorescence imaging proposed in this work owned optimum performance, even exceeding the NIR-IIb fluorescence imaging.

**Fig. 6:**
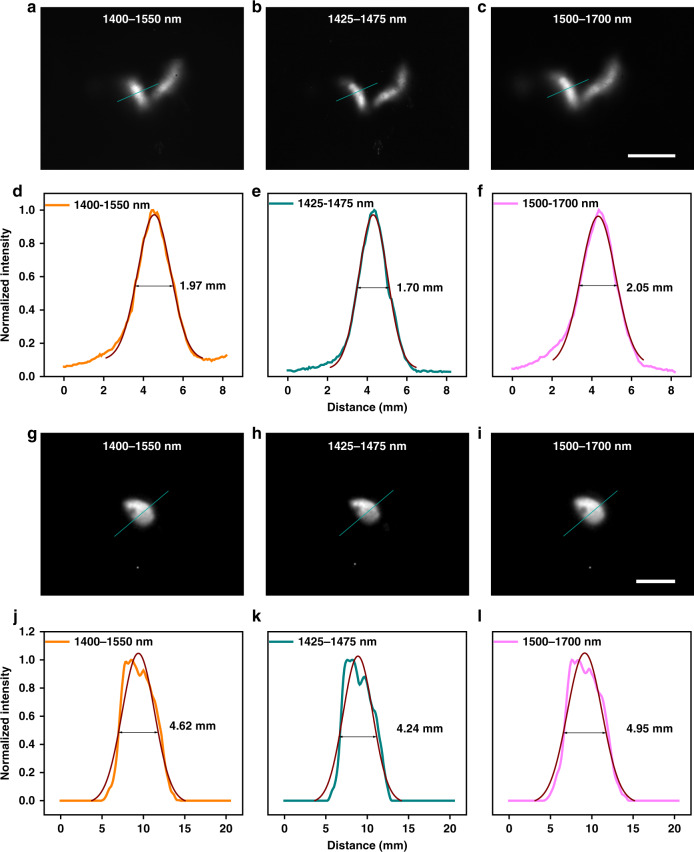
Functional in vivo fluorescence imaging of mice in 1400–1550, 1425–1475, and 1500–1700 nm. The uterine imaging of the same mouse in (**a**) 1400–1550 nm, (**b**) 1425–1475 nm, and (**c**) 1500–1700 nm. **d**–**f** The fluorescence intensity analyses of the right uterus (the indigo lines) in (**a**–**c**). The numbers show the FWHMs. The bladder imaging of the same mouse in (**g**) 1400–1550 nm, (**h**) 1425–1475 nm, and (**i**) 1500–1700 nm. **j**–**l** The fluorescence intensity analyses of the bladder (the indigo lines) in (**g**–**i**). The numbers show the FWHMs. Scale bars: 10 mm.

Deep-penetration fluorescence hysterography gives a promising diagnostic method in uterine anomalies and intrauterine lesions with multiple advantages of noninvasive property, high spatial resolution, and no ionizing radiation. Besides, the bladder is also a hollow organ in the urinary system, that stores and controls urine. The bladder fluorescence imaging benefits precisely monitoring the volume variation which may be related to lower urinary tract symptoms including storage disorder. Figure 6 shows the results of hollow organ imaging and the definitions of imaging in different imaging windows were further compared. After uterine perfusion (1 mg mL^−1^, 200 μL), the bright and specific uterine structure was presented. In uterine imaging (see Fig. 6a–c), the skin, fatty, muscularis, and the organs above, such as the gut and the bladder, were brakes on the photon propagation, leading to a diffuse structural boundary. The image in 1425–1475 nm (see Fig. 6b) gave us the sharpest outline in comparison. The quantitative intensities and the results of Gaussian fitting are displayed in Fig. 6d–f and the FWHMs of the same site were 1.97 mm (1400–1550 nm), 1.70 mm (1425–1475 nm), and 2.05 mm (1500–1700 nm), respectively. Obviously, the highly weakening of background induced by photon scattering could not only enormously increase the image contrast (see Fig. 5) but also improve the spatial resolution of the details deeply buried in the bio-tissue (see Fig. 6). Moreover, the bladder was labeled via intravesical perfusion (1 mg mL^−1^, ~20 μL). As shown in Fig. 6g–l, the imaging quality did not improve with the increase of imaging wavelength, which was not consistent with the general understanding before. Through the skin and muscles, the bladder image showed the clearest contour in 1425–1475 nm with a narrowest fitted diameter of 4.24 mm, while the FWHMs of the bladders in 1400–1550-nm and 1500–1700-nm (NIR-IIb) imaging were 4.62 mm and 4.95 mm. In view of this, the NIR-IIx fluorescence imaging deciphered the deep details in vivo precisely, which held the strong potential to advance the medical imaging in the clinic.

### Large-depth tomography via fluorescence wide-field microscopy around NIR-IIx region

User-friendly fluorescence wide-field microscopy, as a classical technique, was often utilized for cell or tissue slice imaging. In recent years, the imaging window of wide-field microscopy has been shifted into the NIR region to shrink the photon scattering and visualized through the deep tissue ex or in vivo. Nowadays, NIR-II fluorescence wide-field microscopy has succeeded in penetrating ~800 μm in the brain^7,27,28^. However, despite the large imaging depth, the scattering photons and the signal photons outside the focal plane induced background kept the details hidden beneath a veil of “mist”. With the excellent SBR and spatial resolution of the fluorescence imaging around the peak absorption wavelength of water shown above, wide-field microscopy around the NIR-IIx region was believed to possess excellent performance without complex excitation and collection modes.

**Fig. 7:**
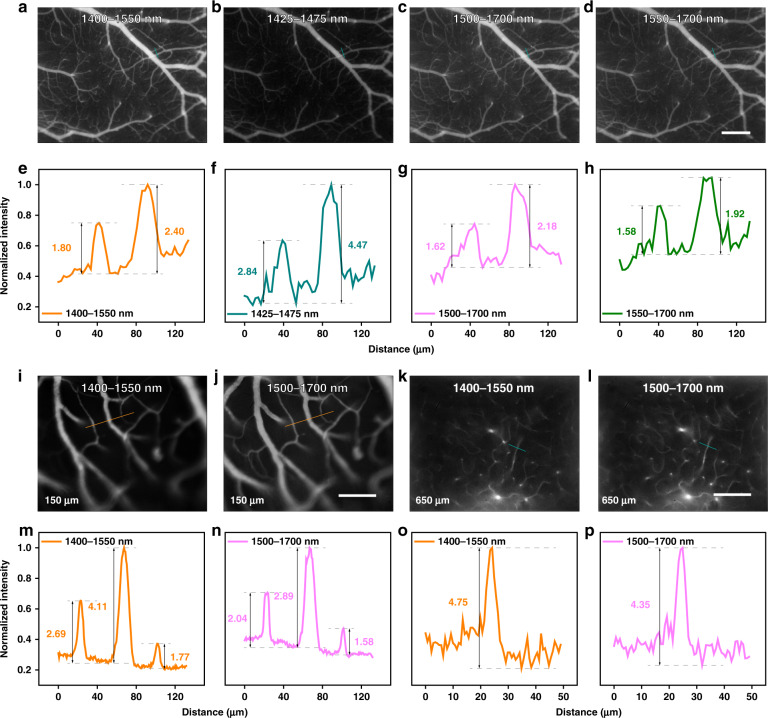

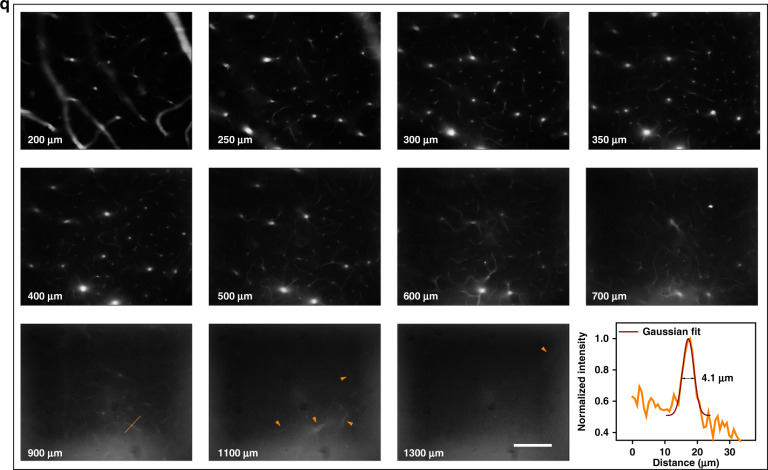
In vivo fluorescence wide-field microscopic imaging beyond 1400 nm. The ×5 microscopic imaging of cerebral vasculature in the same mouse in (**a**) 1400–1550 nm, (**b**) 1425–1475 nm, (**c**) 1500–1700 nm, and (**d**) 1550–1700 nm. Scale bar: 300 μm. **e**–**h** Cross-sectional fluorescence intensity profiles along the indigo lines of the blood vessel in (**a**–**d**). The numbers show the SBRs. The ×25 microscopic imaging of cerebral vasculature in the same mouse at depth of 150 μm in (**i**) 1400–1550 nm and (**j**) 1550–1700 nm. Scale bar: 100 μm. The ×25 microscopic imaging of cerebral vasculature in the same mouse at depth of 650 μm in (**k**) 1400–1550 nm and (**l**) 1550–1700 nm. Scale bar: 100 μm. Cross-sectional fluorescence intensity profiles along (**m**, **n**) the brown lines in (**i**, **j**) and (**o**, **p**) the indigo lines in (**k**, **l**). The numbers show the SBRs. **q** ×25 microscopic imaging of cerebral vasculature in 1400–1550 nm at various depths below the skull and the fluorescence intensity analysis of the blood vessel at the depth of 900 μm (the brown line). The brown arrows show the deep and tiny capillaries. Scale bar: 100 μm.

The ×5 microscopic imaging was conducted with a scan lens (LSM03, Thorlabs) equipped. As shown in Fig. 7a–d, it could be seen that the light absorption by water greatly restrained the background. The quantitative results are presented in Fig. 7e–h, and the SBRs were 1.80 and 2.40 in 1400–1550-nm image, 2.84 and 4.47 in 1425–1475-nm image, 1.62 and 2.18 in 1500–1700-nm image, as well as 1.58 and 1.92 in 1550–1700-nm image, respectively. Inevitably, the strong light absorption would make the loss of useful signals meanwhile, in spite of remarkable contrast improvement. In order to efficiently reduce the signal loss in deep exploring, 1400–1550 nm was then determined as the imaging region for microscopy with larger magnification. The ×25 microscopic images of cerebral vasculature at the depth of 150 μm and 650 μm below the skull are displayed in Fig. 7i–l. The three capillaries in the 1400–1550-nm image all showed higher SBRs of 2.69, 4.11, and 1.77 than those in the NIR-IIb image (2.04, 2.89, and 1.58) at 150 μm (see Fig. 7m, n). Besides, the 650-μm-depth vasculum imaged in 1400–1550 nm possessed the SBR of 4.75, while the NIR-IIb image gave the SBR of 4.35 when measuring the same vessel (see Fig. 7o, p). As shown in Fig. 7q, the images of cortex vessels kept extremely low background within ~500 μm in the brain. At ~900 μm, the vessel network was still clearly visible and the sharp and tiny capillary with an FWHM of just 4.1 μm could be easily distinguished. Beyond 900 μm, the potential white matter might become the obstacle for further visualizing and the image details started to become sparse. At about ~1.3 mm, there still existed recognizable vascular, which represented the largest imaging depth of in vivo NIR-II fluorescence microscopy in the mice brain up to now.

### Off-peak NIR-II fluorescence imaging goes best with 1400 nm long-pass (NIR-IIx + NIR-IIb) collection

Nowadays, many efforts have been made for the design and synthesis of imaging probes with both long-emission wavelength and high brightness, especially for organic probes. The recent researches on the optimization of off-peak NIR-II fluorescence imaging have also expedited the development of bright NIR-II emissive probes. Owing to the considerable emission and the broad emission band, some NIR emissive probes were successfully utilized for NIR-IIb (beyond 1500 nm) fluorescence imaging, which was long-term routinely described as the best NIR-II imaging window resulting from the minimized photon scattering. As a matter of fact, the poorer performance in 1550–1700 nm, compared to the imaging in 1500–1700 nm (NIR-IIb), has made it clear that simply reducing the scattering could not promote the imaging quality (see Fig. 5f, g). Thus, we believed that the improvement of the NIR-IIb window was attributed in large part to the light absorption, in addition, the 1400–1500 nm region, which entirely included the huge absorption peak at ~1450 nm, should not be filtered away during off-peak fluorescence imaging theoretically.

**Fig. 8:**
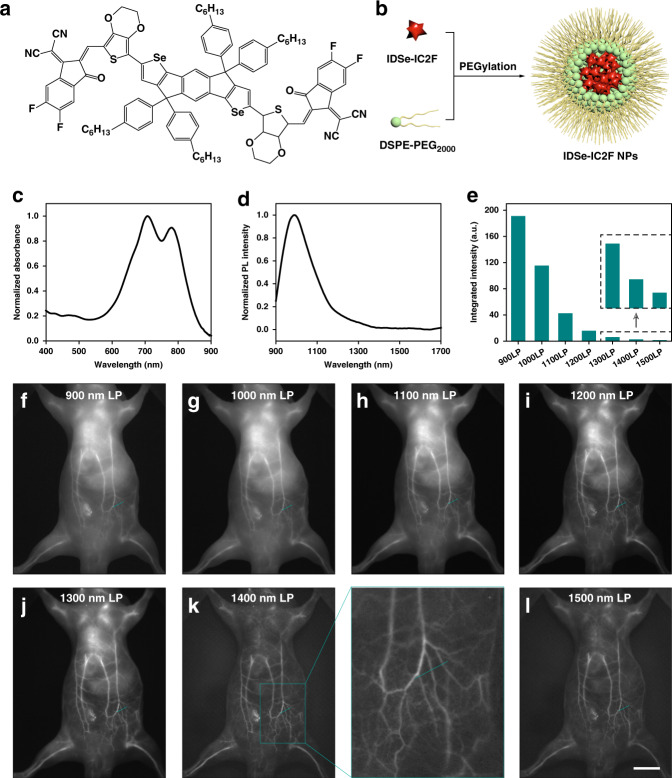

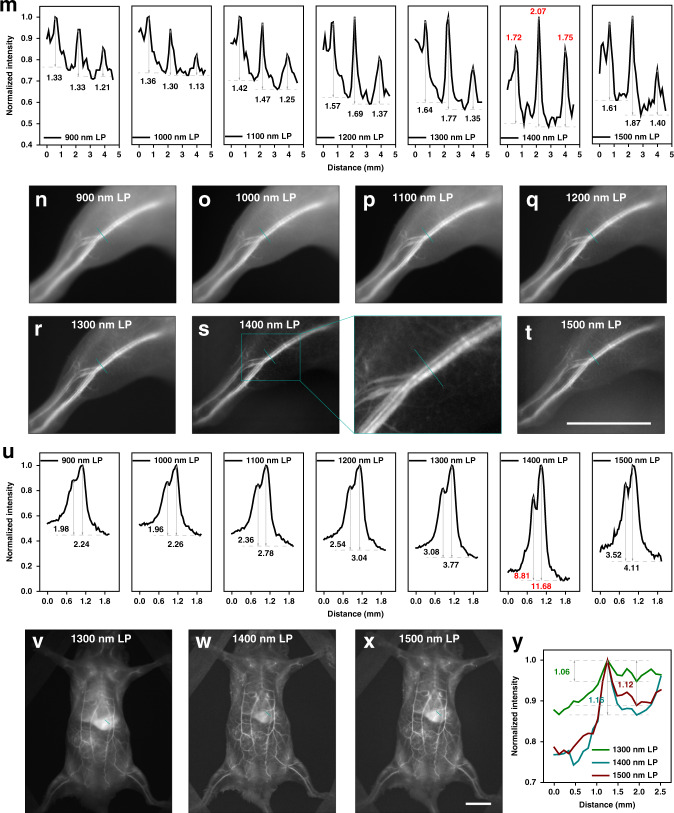
In vivo fluorescence imaging of mice using the emission tail of the probes. **a** The molecular structure of IDSe-IC2F. **b** The schematic illustration of the synthesis of IDSe-IC2F NPs. The normalized (**c**) absorbance and (**d**) emission spectra of IDSe-IC2F NPs. (**e**) The integrated emission intensities in 900–1700, 1000–1700, 1100–1700, 1200–1700, 1300–1700, 1400–1700, and 1500–1700 nm regions. The whole-body fluorescence imaging of the same mouse beyond (**f**) 900 nm, (**g**) 1000 nm, (**h**) 1100 nm, (**i**) 1200 nm, (**j**) 1300 nm, (**k**) 1400 nm, and (**l**) 1500 nm. **m** Cross-sectional fluorescence intensity profiles along the indigo lines of the blood vessel in (**f**–**l**). The numbers show the SBRs. The hind limb fluorescence imaging of the same mouse beyond (**n**) 900 nm, (**o**) 1000 nm, (**p**) 1100 nm, (**q**) 1200 nm, (**r**) 1300 nm, (**s**) 1400 nm, and (**t**) 1500 nm. **u** Cross-sectional fluorescence intensity profiles along the indigo lines of the blood vessel in (**n**–**t**). The numbers show the SBRs. The whole-body fluorescence imaging of the same mouse beyond (**v**) 1300 nm, (**w**) 1400 nm, and (**x**) 1500 nm, after the NPs accumulated in the liver. **y** The fluorescence intensity profiles along the indigo lines of the blood vessel above the liver in (**v**–**x**). The numbers show the SBRs. Scale bars: 10 mm.

As a representative NIR-II dye reported in our previous work^5^, IDSe-IC2F was chosen to explore the optimum imaging window for off-peak NIR-II fluorescence imaging. The molecular structure is shown in Fig. 8a, in which 7,7′‐(4,4,9,9‐tetrakis(4‐hexylphenyl)‐4,9‐dihydro‐s‐indaceno[1,2‐b:5,6‐b′]bis(selenophene)‐2,7‐diyl)bis(2,3‐dihydrothieno[3,4‐b][1,4]dioxine‐5‐carbaldehyde) and 2‐(5,6‐difluoro‐3‐oxo‐2,3‐dihydro‐1H‐inden‐1‐ylidene)malononitrile served as the electron‐donating and ‐withdrawing moieties respectively with a π‐bridge of ethylene dioxothiophene (EDOT) unit. The absorption and emission spectra of the IDSe-IC2F in tetrahydrofuran (THF) were presented in Supplementary Fig. S13a. To improve the aqueous dispersibility, the IDSe-IC2F molecules were encapsulated into nanoparticles (NPs) via PEGylation (Fig. 8b). The TEM and DLS results in Supplementary Fig. S14 show that IDSe-IC2F NPs held a sphere structure with a mean diameter of ~54 nm. The hydrated IDSe-IC2F NPs acted as the typical NIR-II fluorophores with absorption peaks in the NIR-I region and the emission peak in the NIR-II region (Fig. 8c, d and Supplementary Fig. S13b). Besides, the integrated fluorescence intensities in Fig. 8e show the tail emission. After continuous laser irradiation for 120 min, there was almost no decrease in fluorescence intensity of the IDSe-IC2F NPs (see Supplementary Fig. S13c). After intravenous injection of the IDSe-IC2F NPs (1 mg mL^−1^, 200 μL), the whole-body imaging of mice was conducted under the excitation of a 793 nm CW laser, and the corresponding images in 900/1000/1100/1200/1300/1400/1500–1700 nm are shown in Fig. 8f–l. After calculation (see Fig. 8m), the selected three vessels (indigo lines) showed the highest SBRs of 1.72, 2.07 and 1.75 in 1400-nm-LP image, while those in 900-nm-LP, 1000-nm-LP, 1100-nm-LP, 1200-nm-LP, 1300-nm-LP, 1500-nm-LP images were 1.33, 1.33 and 1.21, 1.36, 1.30 and 1.13, 1.42, 1.47 and 1.25, 1.57, 1.69 and 1.37, 1.64, 1.77 and 1.35, 1.61, 1.87 and 1.40, respectively. Besides, the hind limb imaging was further carried out and the background suppression near the absorption peak of water is obvious in Fig. 8n–t. The SBRs of the selected two vessels reached the maximum of 8.81 and 11.68 in 1400–1700-nm imaging (Fig. 8u). It could be concluded that the performance of NIR-IIx + NIR-IIb (1400–1700 nm) imaging surpassed the NIR-IIb imaging since the NIR-IIx signals did a positive contribution with only slight background added. Moreover, the quality of 900-nm-LP fluorescence imaging was no worse than that of the 1000-nm-LP fluorescence imaging, which once again demonstrated that 900–1000 nm should not be excluded from the NIR-II region. In the images at 2 h post injection, the liver was lit up (Fig. 8v–x), since substantial IDSe-IC2F NPs in blood accumulated in the liver. As shown in Fig. 8y, the NIR-IIx + NIR-IIb fluorescence imaging could present the targeted vessels above the liver with a higher SBR of 1.16 while the SBRs of the same vessel were 1.06 and 1.12 in fluorescence imaging beyond 1300 nm and 1500 nm. Thus, the results showed clearly that the extra collection of 1400–1500 nm was conductive to the precise deciphering and the NIR-IIx + NIR-IIb fluorescence detection would bring new ideas for off-peak fluorescence imaging and even the imaging-guided surgery in the clinic.

## Discussion

Previous viewpoints have always emphasized that the decrescent photon scattering brought by the wavelength increase mainly contributed to the ameliorated performance of NIR-II fluorescence imaging but the light absorption peak by water has long been kept off. Admittedly, the excessive absorption of the excitation light might induce serious photo-thermal damage. However, the intensity of excited fluorescence is always far less than that of excitation light, thus the induced thermal effect could be ignored when the emission region is located near the absorption peak. On the other hand, it is generally accepted that light absorption would also significantly decrease the useful signals. As for point detection (common in scanning microscopy), the excited pointed signals are collected into the detector (such as photomultiplier tube) and then the ballistic and diffused signals are both restructured on one pixel. On account of the effective signal collection, the absorption enhancement is usually undesirable in this situation. Notwithstanding, the signal diffusion is influential in the 2D area detection and our simulation results revealed that the moderate light absorption by the bio-tissue could attenuate the scattering photons with a long optical path and thus restrain the image background in the 2D detection. Based on this, we defined the promising regions of 1400–1500, 1700–1880, and 2080–2340 nm as NIR-IIx, NIR-IIc, and NIR-III windows, and the NIR-III imaging was believed to provide the best imaging quality in the whole NIR region. The lack of suitable imaging probes and commercialized IR detectors limits the in vivo fluorescence imaging experiments beyond 1700 nm at present. We look forward to the novel fluorophores, such as long-wavelength-emitting QDs, and the highly efficient detection technique, such as the extended InGaAs camera, in the future eagerly.

It should be noted that, as for some superficial tissues labeling without deep penetration, the brightness of the labeling probes should be a major concern. Recent work has revealed that the intraperitoneal tumor nodules labeled by a kind of bright NIR-I emissive aggregation-induced emission (AIE) agents exhibited a high SBR (tumor-to-surrounding normal intestine) value of 48.5 ± 5.6 after the mouse abdomen was opened^49^. Thus, the superiority of the NIR-II imaging should be discussed when visualizing the detailed signals buried in the more complicated background. After verification of in vivo imaging, we believed that fluorescence imaging in the whole 900–1880 nm region showed desirable imaging quality resulting from the positive influence of incremental light absorption and decrescent photon scattering, compared with the imaging in the NIR-I window, the NIR-II region should be defined as 900–1880 nm, therefore. Besides, the promising performance of fluorescence imaging beyond 1300 nm, which was generally considered as NIR-IIa fluorescence imaging, further confirmed the dominance of light absorption. NIR-IIx region, which was defined as 1400–1500 nm here, contained the intense absorption at ~1450 nm and was proved to own the optimum imaging potential, even exceeding the NIR-IIb region. When the emission is strong enough to resist the absorption loss, the collection within the NIR-IIx region should be a recommendable option.

In addition to the QDs and the mentioned organic nanoprobes in this work, there have been many other kinds of excellent NIR-II emissive probes developed for deep imaging, such as small-molecule fluorescence dyes, polymer dots, AIE dots, rare-earth-doped nanoparticles, etc. Among them, Tm^3+^-doped rare-earth NPs possess great potential as a kind of high-efficiency downconversion fluorophores with sharp NIR-IIx emission. With the large and broad spectral absorption property, the polymer dots always exhibit remarkable fluorescence and could be regarded as a strong candidate for NIR-IIx imaging. Overall, the absolute emission ability in the NIR-IIx region determined whether the optical probes could be directly utilized in the NIR-IIx imaging. As for the probes with limited peak emission wavelength at present, the off-peak NIR-II fluorescence imaging was an efficient compromise. There have been some bright fluorophores developed for off-peak NIR-IIa or NIR-IIb fluorescence imaging, however, the NIR-IIx region was neglected. The LP usage with NIR-IIx + NIR-IIb detection (1400 nm LP) was proved to enhance image contrast. Adding the NIR-IIx collection, meanwhile, relaxed the requirements of the fluorophores, which benefited the off-peak fluorescence detection. The conclusion might guide the future off-peak fluorescence bioimaging and promote the development of the NIR-II fluorescent probes.

The poor sectioning strength of wide-field microscopy was attributed to the interference by the scattering and defocus signals. When shifting the imaging window around the NIR-IIx region, the image contrast was remarkably promoted. The performance of NIR-IIx fluorescence wide-field tomography was even comparable with that of the scanning microscopy, while the latter usually possessed a more complex optical setup and lower temporal resolution. We believe an ingenious and simplified technique for sectioning imaging was proposed here and more imaging agents emitting around the NIR-IIx region in the future can be anticipated.

Finally, it should be pointed out that besides fluorescence imaging, the interactions between light and bio-tissue should be reconsidered in other advanced imaging techniques. For instance, aiming at deep penetration, the excitation wavelength of photoacoustic imaging has been extended to the NIR-II region. However, when photoacoustic imaging was performed with the assistance of some exogenous probes, the strong absorption of bio-tissues themselves would deplete the NIR-II excitation on the target. Recent work^50^ has revealed that the depth of NIR-II (1150–1700 nm) light excited photoacoustic imaging was limited due to the significantly attenuated photon energy caused by the increased optical absorption of water in the tissue. In nonlinear optical microscopy, multiphoton excitation of fluorophores in tissue depends on the focusing capability of excitation light. The NIR-III window theoretically minimizes the photon scattering. Compared with the NIR-IIx window, the light absorption in the NIR-III region is relatively lower. Besides, the scanning point-excitation mode could relieve the photo-thermal injury to a certain extent, rather than the area-excitation mode. Thus, we believe our proposal of the NIR-III window could drive the development of nonlinear optical microscopy with further deepening penetration. Considering the spectral properties of the developing nonlinear optical probes at present, the four-photon microscopy and third-harmonic generation microscopy excited by the NIR-III light were much awaited.

## Materials and methods

### Simulation of the NIR photon propagation in biological tissues

The Monte Carlo method was utilized to simulate the propagation of photons in biological tissues. The total thickness of the tissue used in the simulation was 4 mm, with length and width of 100 mm and 100 mm, respectively. We set the refractive index of the tissue as 1.37, the scattering anisotropy factor as 0.9, and the refractive index of air as 1. In the simulation, the absorption coefficient of water was considered as the tissue absorption coefficient since the absorptions by other components in tissues were much slighter compared with that by water^51^, and the reduced scattering coefficient was calculated using the following formula: *μ*_s_*’* = 1.5 *λ*^−1^ (mm^−1^). The fluorescence signal source was a line with a length of 40 mm, a width of 1 mm, and a depth of 1 mm in the tissue, which would emit a total of 10^6^ photons in random directions. The detection plane with 400 × 400 pixels was 1 mm parallel above the tissue surface. After successfully escaping from the tissue, a signal photon fell on a certain pixel on the detection surface. A simulated image was obtained by integrating the signals of all outgoing photons. Some point light sources with random emitting directions were added at random locations in the tissue as noise. There were 10^6^ noise photons in each simulation.

### Synthesis and PEGylation of the PbS/CdS quantum dots

For all details about synthesis and PEGylation of the CSQDs, please see the Supplementary Information.

### Animal preparation

All experimental procedures were approved by Animal Use and Care Committee at Zhejiang University. All the experimental animals involved, including BALB/c nude mice (~20 g) and Institute of Cancer Research (ICR) mice (~20 g), were provided from the SLAC Laboratory Animal Co. Ltd. (Shanghai, China) and fed with water and food with a normal 12 h light/dark cycle.

### Functional fluorescence imaging in vivo

All the experiments and operations on mice were conducted under anesthesia. After intravenous injection of PbS/CdS CSQDs (1 mg mL^−1^, 200 μL) or IDSe-IC2F NPs (1 mg mL^−1^, 200 μL), the whole-body vessel imaging was performed under the expanded 793 nm CW laser excitation. As shown in Supplementary Fig. S15, the NIR-II signals were collected using a fixed focus lens with NIR antireflection (TKL35, focal length of 35 mm, Tekwin, China), purified by different filters (The 900-, 1000-, 1100-, 1200-, 1300-, 1400-, and 1500-nm long-pass filters were purchased from Thorlabs; The 1450 nm band-pass filter, 1550-nm long-pass filter, 1100 nm and 1300 nm short-pass filters were purchased from Edmund Optics; The 1550 nm short-pass filter was custom-built), and eventually imaged on the electronic-cooling InGaAs camera (SD640, Tekwin, China). Next, the image distance was elongated to shrink the line field of view and thus the hind limb could be clearly presented. As for the imaging in Fig. 3, the power densities of the excitation were ~2, ~2, ~12, and ~12 mW cm^−2^ in Fig. 3d, e, g, and h, respectively. As for the imaging in Fig. 4, the power densities of the excitation were ~3, ~30, ~15, ~37, ~60, and ~60 mW cm^−2^ in Fig. 4d, e, g, h, j, and k, respectively. As for the imaging in Fig. 5, the power densities of the excitation were ~40, ~40, ~20, ~20, ~40, ~40, ~40, and ~40 mW cm^−2^ in Fig. 5d, e, f, g, j, k, l, and m, respectively. As for the imaging in Fig. 6, the power densities of the excitation were ~30, ~30, ~30, ~10, ~10, and ~10 mW cm^−2^ in Fig. 6a, b, c, g, h, and i, respectively. As for the imaging in Fig. 8, the power densities of the excitation were ~1, ~2, ~30, ~55, ~75, ~110, ~110 , ~1.5, ~2, ~40, ~70, ~100, ~150, ~150, ~75, ~110, and ~110 mW cm^−2^ in Fig. 8f, g, h, i, j, k, l, n, o, p, q, r, s, t, v, w, and x, respectively. As for uterine imaging, the 1450-PbS/CdS CSQDs (1 mg mL^−1^, 200 μL) were infused into the uterine cavity via a 26 G catheter. The mice with the uterus labeled were then placed in the macro imaging system and the bright NIR-II fluorescence from the uterus could be further detected. To achieve NIR-II fluorescence cystography, the 1450-PbS/CdS CSQDs (1 mg mL^−1^, ~20 μL) were retrogradely injected into the bladder of mice with another 26 G catheter, then the bladder-labeled mice were moved to the macro imaging system.

### Wide-field microscopic fluorescence imaging in vivo

The skulls of the ICR mice were opened before imaging and the round thin coverslips were mounted on the brain. The treated mice were fixed after injected with the 1450-PbS/CdS QDs (5 mg mL^−1^, ~200 μL). As shown in Supplementary Fig. S16, the 793 nm CW laser was introduced into the upright NIR-II fluorescence microscope (NIR II-MS, Sunny Optical Technology). The expanded 793 nm light was reflected by the dichroic mirror, passed through the objective, and evenly excited the fluorophores covered in the bio-tissue. The fluorescence signals were collected by the objective, transmitted through the dichroic mirror and equipped filters, and were focused by the tube lens onto the imaging detector. Different magnifications could be obtained by changing the objective.

### The measurement of emission spectra

The excitation light was introduced into the lab-built measurement system shown in Supplementary Fig. S17. The emission signals were collected by a NIR objective and finally detected by a NIR spectrometer (NIR2200, Ideaoptics Instruments, China).

### Statistical analysis

Quantitative analysis of intensity was conducted by Image J software (National Institutes of Health, USA). Origin Pro software (OriginLab Company, USA) was used to generate the graphs. The data in Fig. 2 are presented as mean ± standard errors of the mean.

## Supplementary information

Supplementary Information
