# Supplementary material for: Perfecting and extending the near-infrared imaging window
Source: Light Sci Appl. 2021 Sep 24;10:197. doi: 10.1038/s41377-021-00628-0 (PMC8463572; doi:10.1038/s41377-021-00628-0)
Supplement: Supplementary file 1 — Supplementary Information [file 41377_2021_628_MOESM1_ESM.pdf]

Supplementary Information for

Perfecting and extending the near-infrared imaging window

Zhe Feng[1, 2]†, Tao Tang[3]†, Tianxiang Wu[1], Xiaoming Yu[4], Yuhuang Zhang[1, 2], Meng Wang[3], Junyan Zheng[4], Yanyun Ying[4], Siyi Chen[1], Jing Zhou[1], Xiaoxiao Fan[1], Dan Zhang[4], Shengliang Li[5], Mingxi Zhang[3]* and Jun Qian[1, 2]*

[1]*State Key Laboratory of Modern Optical Instrumentations, Centre for Optical and Electromagnetic Research, College of Optical Science and Engineering, International Research Center for Advanced Photonics, Zhejiang University, Hangzhou 310058, China*

[2]*Intelligent Optics & Photonics Research Center, Jiaxing Institute of Zhejiang University, Jiaxing 314000, Zhejiang Province, China*

[3]*State Key Laboratory of Advanced Technology for Materials Synthesis and Processing, Wuhan University of Technology, Wuhan 430070, China*

[4]*Key Laboratory of Reproductive Genetics (Ministry of Education), Department of Reproductive Endocrinology, Women's Hospital, Zhejiang University School of Medicine, Hangzhou 310006, China*

[5]*College of Pharmaceutical Sciences, Soochow University, Suzhou 215123, China*

†*These authors contributed equally.*

**Correspondence and requests for materials should be addressed to J.Q. (E-mail: qianjun@zju.edu.cn) or to M.Z. (E-mail: mxzhang@whut.edu.cn)*

Supplementary methods

Simulation of the NIR photon propagation through the subcutaneous adipose tissue

V. V. Tuchin et al (*Appl. Optics* **32**, 3531-3540, 1993) gave the absorption coefficient (Fig. S3a) and reduced scattering coefficient ($\mu_s' = 25.51\lambda^{-0.12}$) of adipose in 2004. The tissue depth was set as 2 mm. Other parameters were consistent with the simulation in Fig. 2.

Materials

$PbCl_2$, CdO, and sulfur powder were purchased from Alfa. Oleylamine, oleic acid, 1-octadecene (ODE), tetrachloroethylene (TCE), poly(acrylic acid) (MW~1,800), N, N'-dicyclohexylcarbodiimide (DCC), N-(3-dimethylaminopropyl)-N′-ethylcarbodiimide hydrochloride (EDC), 2-(N-morpholino)ethanesulfonic acid (MES) were purchased from Sigma-Aldrich. Phosphate-buffered saline (PBS) was purchased from Hyclone. mPEG-amine (MW~5K) was purchased from Laysan Bio. 8-Arm PEG-amine (MW~40K) was purchased from Advanced BioChemicals. Other reagents were of analytical grade. Deionized ultra-filtered (DIUF) water was used in all needed experiments. The deuterium oxide was purchased from J&K Scientific Ltd.

Synthesis of PbS/CdS QDs

Sulfur precursor solution was prepared by mixing 0.08 g of sulfur powder, and 7.5 mL of oleylamine in a two-neck flask at 120°C under argon for 30 min. Lead precursor solution 0.834 g of $PbCl_2$ and 7.5 mL of oleylamine in a three-neck flask was degassed for 30 min under argon and heated to 45-150°C (depending on the desired particle size). 2.25 mL of the sulfur precursor solution was injected into the Pb precursor solution under stirring. When the desired growth time was reached (typically 3-60 min), the reaction was quenched by adding 10 mL of cold hexane. The products were collected by centrifugation and re-suspended in 10 mL hexane/20 mL oleic acid. The mixture was agitated

for 10 min to remove excess sulfur from the products. The QDs were precipitated via centrifugation. This precipitation procedure with oleic acid was repeated 3 times until the supernatant was colorless. After centrifugation of the suspension and decantation of the supernatant, the QDs were re-suspended in toluene/ODE.

The PbS/CdS CSQDs were synthesized via cation-exchange procedure. CdO (0.6 g, 4.6 mmol), oleic acid (4 mL), and ODE (15 mL) were heated to 200 °C, purged with Ar, and then cooled down to 100 °C. 5 mL of previously prepared PbS QDs suspended in Toluene/ODE was bubbled with Ar for 5 min, and then injected into the Cd precursor. The reaction flask was quenched with stopping stirring and heating directly after the growth reaction was conducted at 100 °C for 20-100 min. PbS/CdS CSQDs were precipitated with ethanol and then re-dispersed in hexane.

Modification of CSQDs with oleyamine-branched polyacrylic acid (OPA)

0.9 g of poly (acrylic acid) powder (average MW~1800) and 1.56 g DCC were transferred into a round-bottom flask. 10 mL of DMF was added to dissolve the mixture. About 1.2 mL of oleylamine was added dropwise into the reaction flask. The molar ratio of oleylamine to poly (acrylic acid) is 30%. The solution was stirred overnight. 50 mL of 0.5 M HCl was added to the reaction solution. The precipitate was separated by centrifugation and re-dissolved in 3 mL methanol solution. And then 20 mL 1 M HCl was added to the solution. The precipitate was separated by centrifugation. This procedure was repeated at least 5 times. The precipitate was dissolved in 5 mL chloroform and washed by 10 mL 1 M HCl. The organic phase was collected and dried over by anhydrous Na_2SO_4. The chloroform was removed under vacuum, and the white solid was collected. The average MW of OPA was ~ 3000 determined by gel permeation chromatography.

For surface modification, as-synthesized PbS/CdS QDs (5.0 mg) were dissolved in 2.0 mL

chloroform containing 15 mg of OPA. The mixture was stirred at room temperature for 30 min and the solvent was removed under vacuum by a rotary evaporator. The residue was then dissolved in 2 mL of 50 mM sodium carbonate solution under the sonication. The CSQDs were precipitated with ultracentrifuge at 750,00 rpm for 1 h. The purified product was dissolved in pH 8.5 MES buffer (0.01 M) and stored at 4 °C.

PEGylation of PbS/CdS QDs

OPA-modified PbS/CdS QDs (5 mg) were dissolved in 20 mL pH 8.5 MES buffer (0.01 M). 15 mg of mPEG-amine (MW ~ 5K) and 5 mg of 8-Arm PEG-amine (MW ~ 40 K) with a molar ratio of 24: 1 were dissolved 1 mL MES, and gradually added to QDs dispersion with stirring. 10 mg of EDC was dissolved in 500 μL MES, and gradually added to QDs dispersion with stirring. The mixture was stirred at room temperature overnight. The PEGylated QDs were purified by 100 kDa filter, and washed five times with 1x PBS to remove excess reactants. The purified product was dissolved in 1x PBS and stored at 4 °C.

Supplementary figures

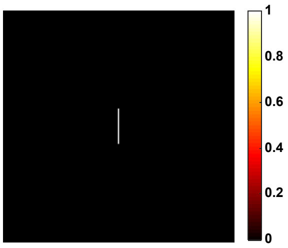

Fig. S1 | The line sample in simulation.

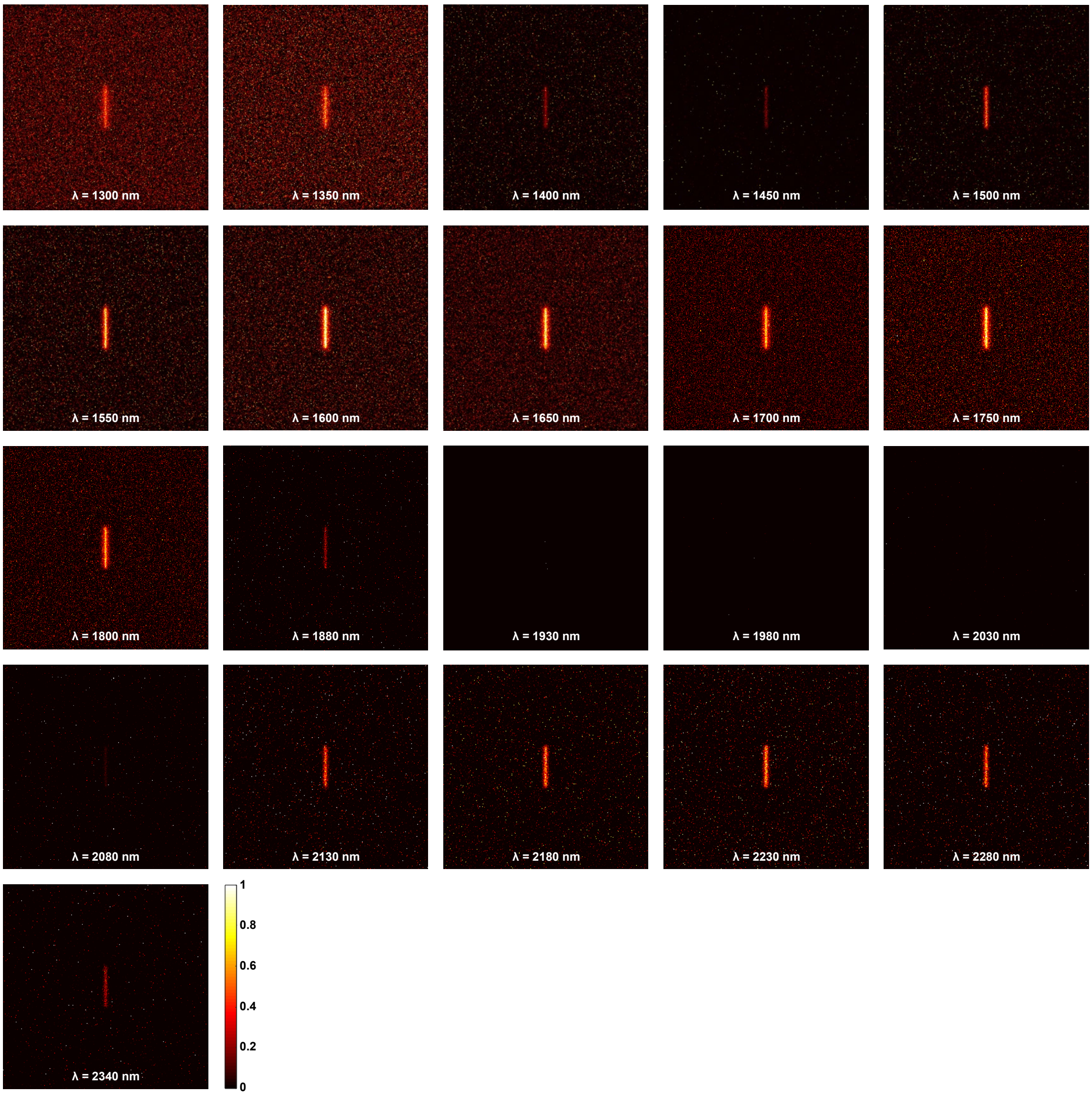

Fig. S2 | The simulation results at specific wavelengths.

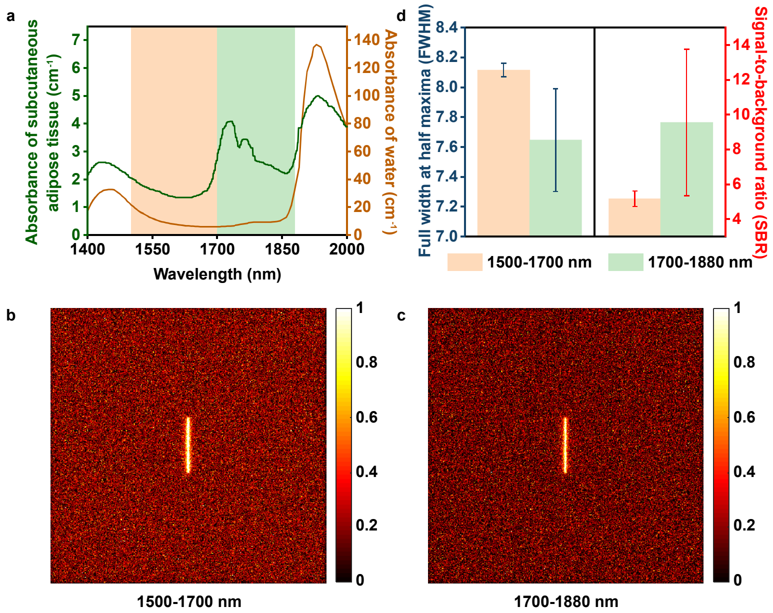

Fig. S3 | The simulation results in the NIR-IIb and the NIR-IIc window. (a) The absorbance of subcutaneous adipose tissue (*Opt. Spectrosc.* **99**, 836-842, 2005) and water (*Appl. Optics* **32**, 3531-3540, 1993). The simulation results of the (b) NIR-IIb and (c) NIR-IIc imaging in the adipose tissue. (d) The calculated FWHMs and SBRs of the simulated samples.

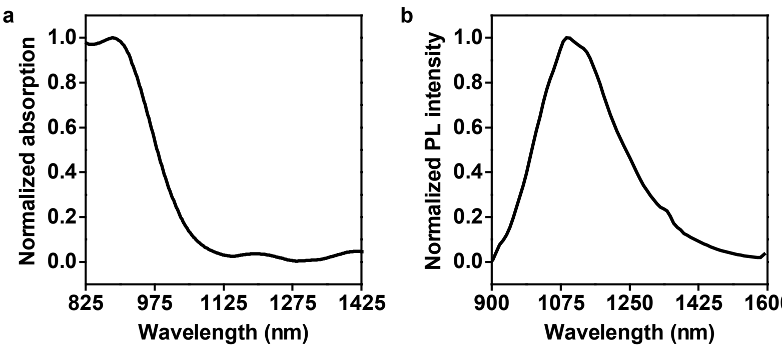

Fig. S4 | The normalized **(a)** absorption (825-1425 nm) and **(b)** PL spectra of 1100-PbS/CdS QDs in tetrachloroethylene.

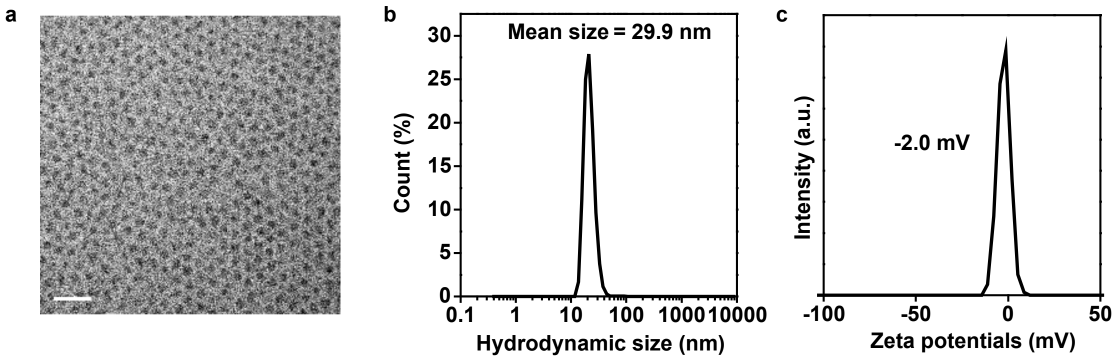

Fig. S5 | (a) The TEM image of as-prepared 1100-PbS/CdS QDs. Scale bar, 20 nm. The **(b)**

hydrodynamic light scattering **(c)** and zeta potential results of the PEGylated 1100-PbS/CdS QDs.

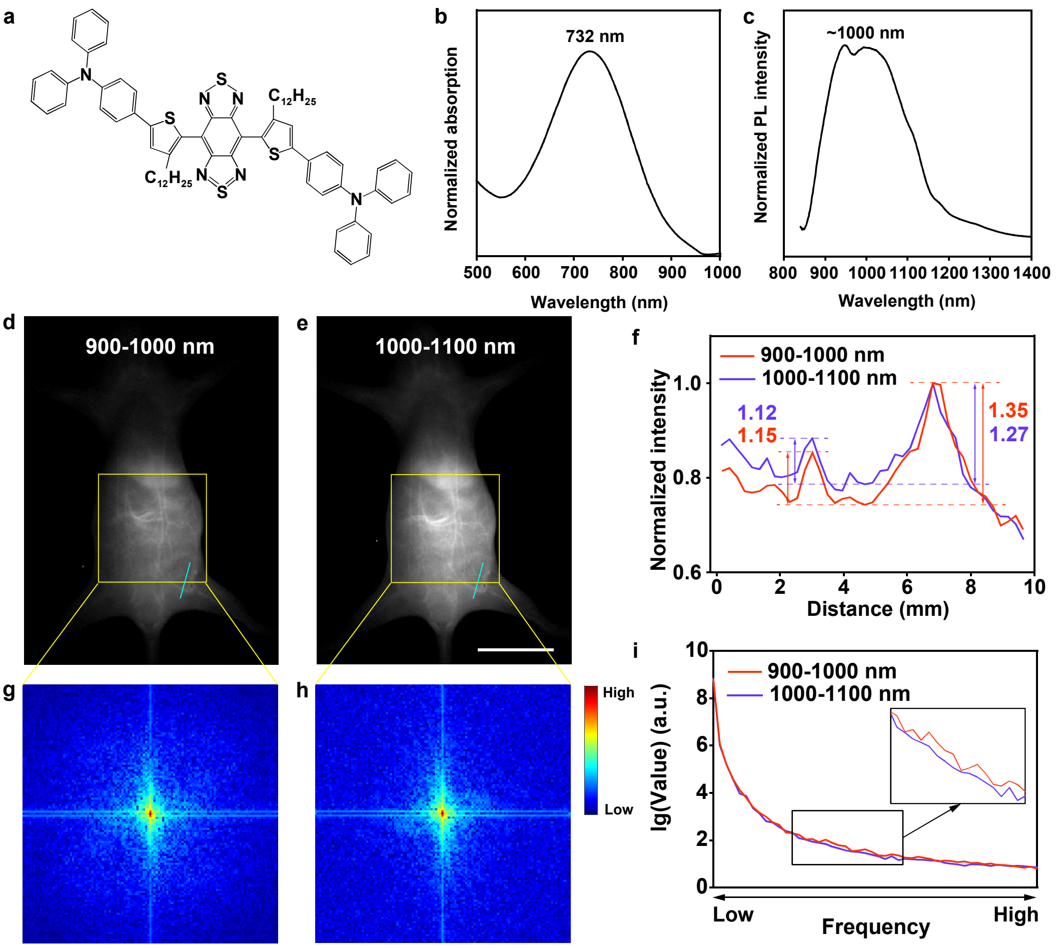

Fig. S6 | Comparison between the 900-1000-nm imaging and the 1000-1100-nm imaging of mice using the reported probes emitting at ~1000 nm (*ACS Nano* 2020, 14, 10, 14228–14239). **(a)** The molecular structure of TT1-oCB. The normalized **(b)** absorption and **(c)** PL spectra of TT1-oCB NPs. The whole-body fluorescence imaging of the same mouse after intravenous injection of TT1-oCB NPs (1 mg mL^{-1}, 200 μL) in **(d)** 900-1000 nm and **(e)** 1000-1100 nm. Scale bar, 20 mm. **(f)** Cross-sectional fluorescence intensity profiles along the indigo lines of the blood vessel in Fig. S6d, e. The numbers show the SBRs. **(g-h)** The fast Fourier transform (FFT) images of the areas selected by the yellow squares in Fig. S6d, e. **(i)** The spatial frequency distributions of Fig. S6g, h.

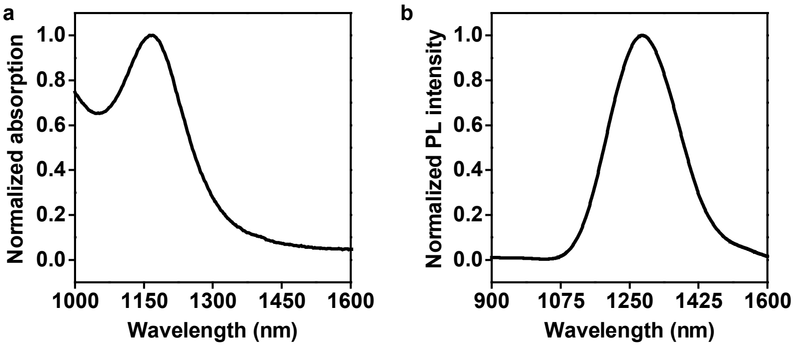

Fig. S7 | The normalized **(a)** absorption (1000-1600 nm) and **(b)** PL spectra of 1300-PbS/CdS QDs in tetrachloroethylene.

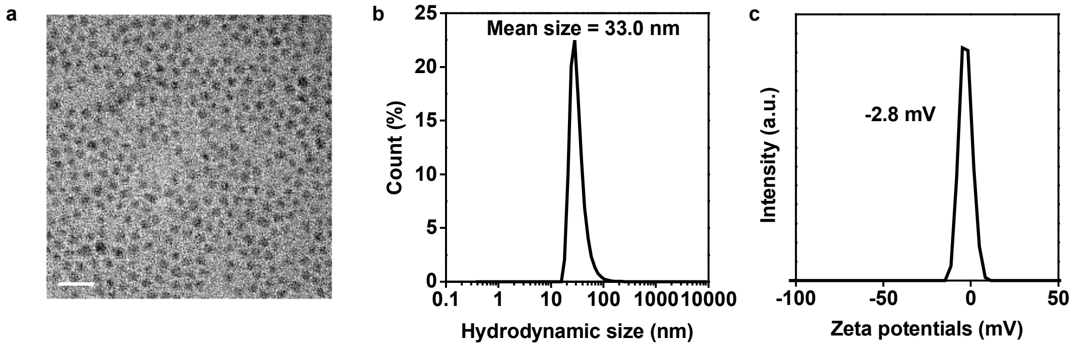

Fig. S8 | (a) The TEM image of as-prepared 1300-PbS/CdS QDs. Scale bar, 20 nm. The **(b)** hydrodynamic light scattering and **(c)** zeta potential results of the PEGylated 1300-PbS/CdS QDs.

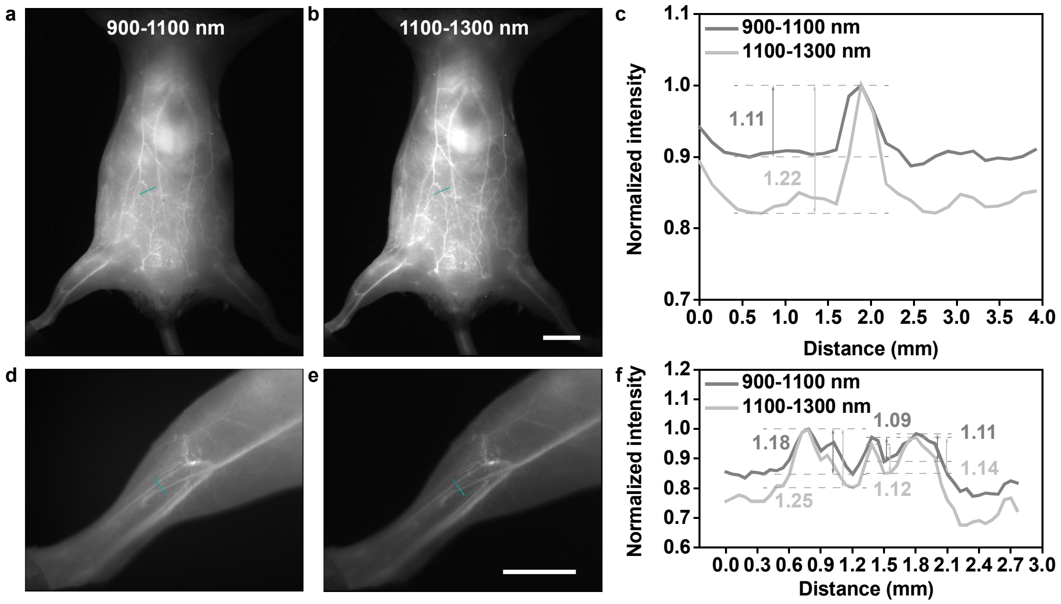

Fig. S9 | Comparison of *in vivo* fluorescence imaging of mice in 900-1100 nm and 1100-1300 nm. The whole-body imaging of of the same mouse in **(a)** 900-1100 nm and **(b)** 1100-1300 nm. **(c)** Cross-sectional fluorescence intensity profiles along the indigo lines of the blood vessel in Fig. S9a, b. The numbers show the SBRs. The hind limb imaging of the same mouse in **(d)** 900-1100 nm and **(e)** 1100-1300 nm. **(f)** Cross-sectional fluorescence intensity profiles along the indigo lines of the blood vessel in Fig. S9d, e. The numbers show the SBRs. Scale bars, 10 mm.

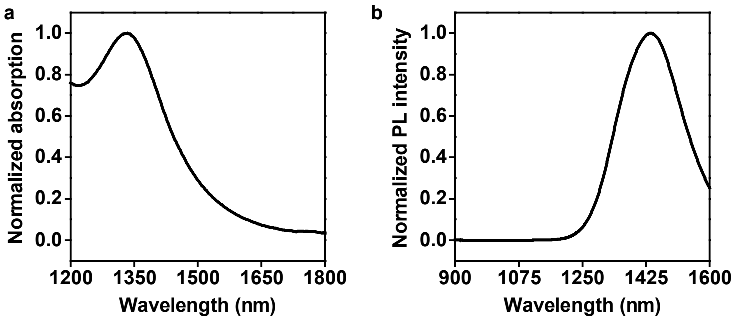

Fig. S10 | The normalized **(a)** absorption (1200-1800 nm) and **(b)** PL spectra of 1450-PbS/CdS QDs in tetrachloroethylene.

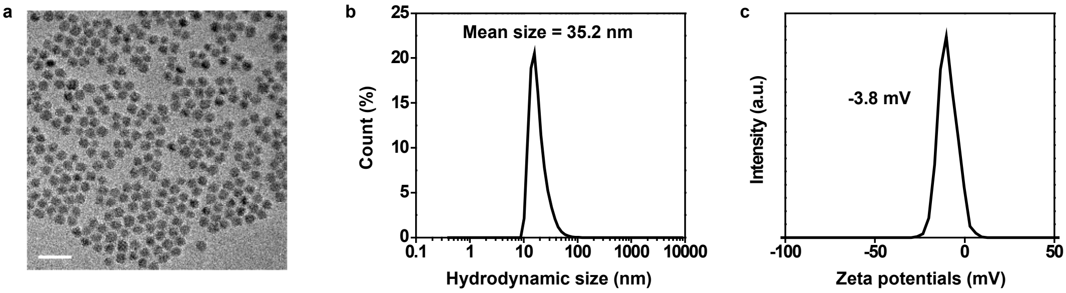

Fig. S11 | (a) The TEM image of as-prepared 1450-PbS/CdS QDs. Scale bar, 20 nm. The **(b)** hydrodynamic light scattering and **(c)** zeta potential results of the PEGylated 1450-PbS/CdS QDs.

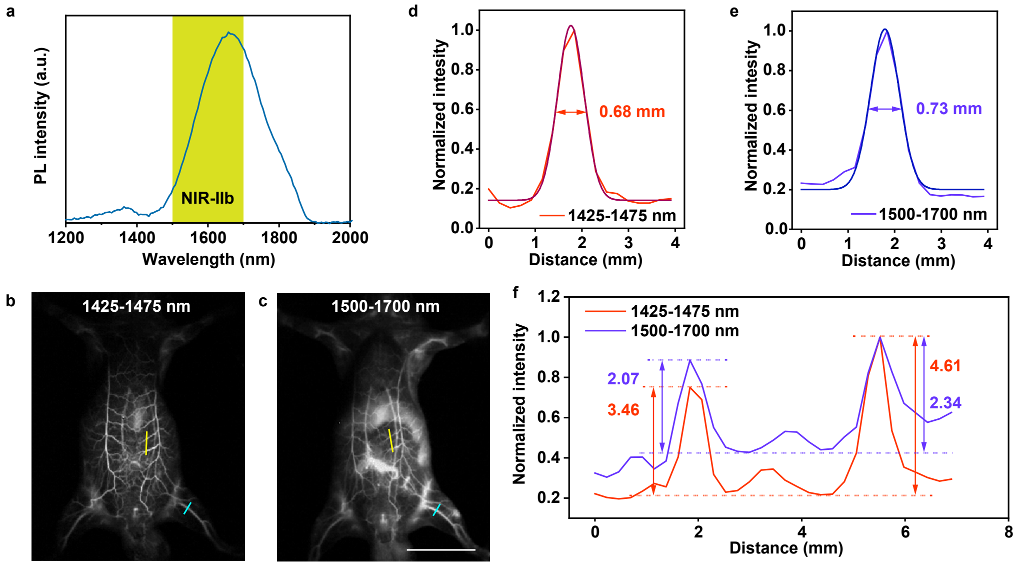

Fig. S12 | Comparison between the 1425-1475-nm imaging and the 1500-1700-nm imaging of mice using the 1600-PbS/CdS QDs, which were reported as a kind of bright NIR-IIb probes (*Proc. Natl. Acad. Sci. USA*. 2018, 115, 6590-6595)**. (a)** The PL spectrum of the 1600-PbS/CdS QDs. The whole-body fluorescence imaging of the same mouse in **(b)** 1425-1475 nm and **(c)** 1500-1700 nm after intravenous injection of 1600-PbS/CdS QDs (1 mg mL^{-1}, 200 µL). Scale bar, 20 mm. **(d-e)** The FWHM analyses of the same vessel selected by the indigo lines in Fig. S12b, c. **(f)** Cross-

sectional fluorescence intensity profiles along the yellow lines of the blood vessel in Fig. 12b, c. The numbers in Fig. S12f show the SBRs.

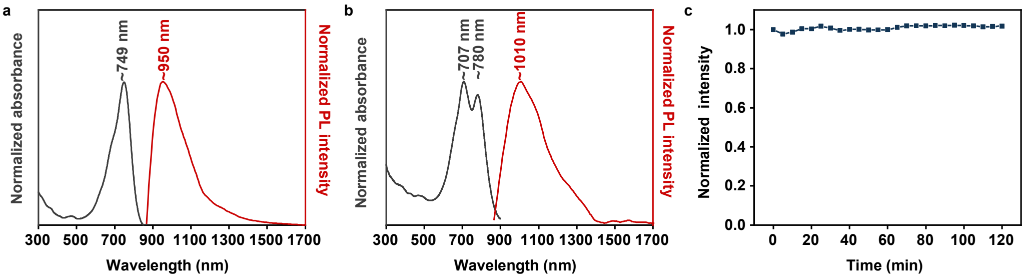

Fig. S13 | The normalized absorption and PL spectra of **(a)** the IDSe-IC2F molecules in THF and **(b)** the IDSe-IC2F NPs in water. **(c)** The photostability test of the IDSe-IC2F NPs under the laser irradiation of 793 nm.

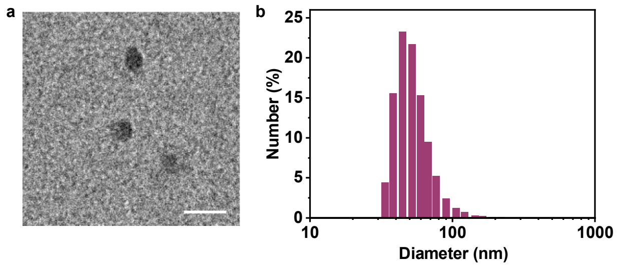

Fig. S14 | (a) The TEM image of the IDSe-IC2F NPs. Scale bar, 50 nm. **(b)** The DLS results of the IDSe-IC2F NPs in water.

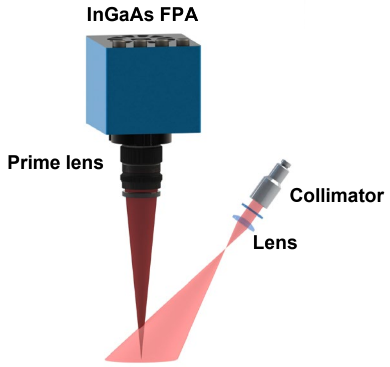

Fig. S15 | The schematic illustration of the macro NIR-II fluorescence imaging system.

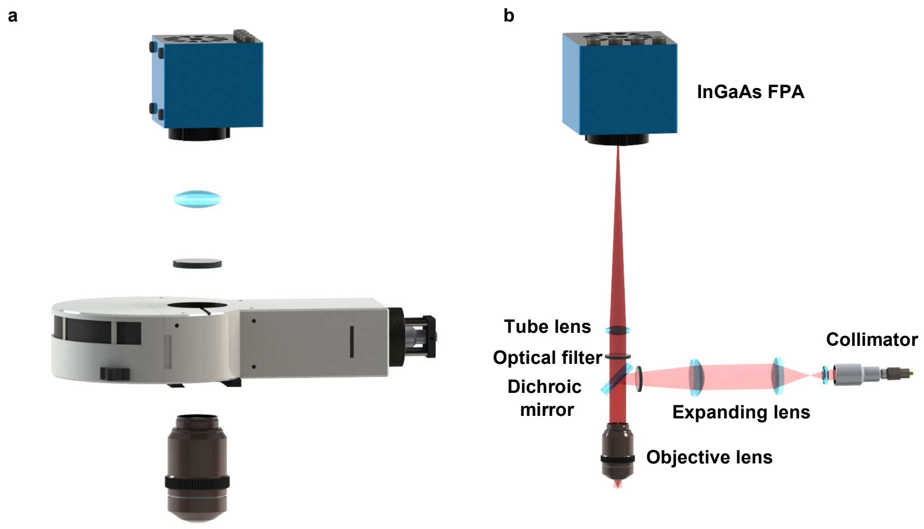

Fig. S16 | The **(a)** optical setup and **(b)** light-path diagram of the microscopic NIR-II fluorescence imaging system.

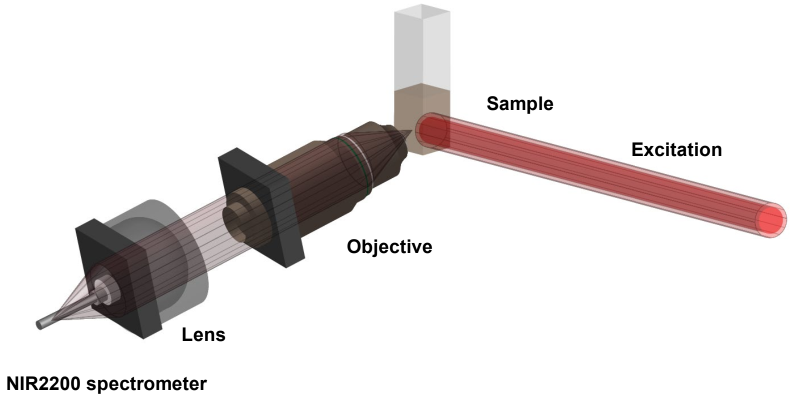

Fig. S17 | The spectra measurement system based on a NIR spectrometer.
